# First-line Treatment With Bendamustine and Rituximab for Old and Frail Patients With Aggressive Lymphoma: Results of the B-R-ENDA Trial

**DOI:** 10.1097/HS9.0000000000000808

**Published:** 2022-12-01

**Authors:** Friederike Braulke, Florian Zettl, Marita Ziepert, Andreas Viardot, Christoph Kahl, Gabriele Prange-Krex, Agnieszka Korfel, Martin Dreyling, Alexander Bott, Ulrich Wedding, Dietmar Reichert, Maike de Wit, Frank Hartmann, Viola Poeschel, Norbert Schmitz, Mathias Witzens-Harig, Wolfram Klapper, Andreas Rosenwald, Gerald Wulf, Bettina Altmann, Lorenz Trümper

**Affiliations:** 1 Department of Hematology and Medical Oncology, University Medical Center Goettingen, Germany; current address: Comprehensive Cancer Center, University Medical Center Goettingen, Germany; 2 Department of Hematology and Oncology, Klinikum Traunstein, Germany; 3 Institute for Medical Informatics, Statistics and Epidemiology (IMISE), University of Leipzig, Germany; 4 Department of Internal Medicine III, University Hospital Ulm, Germany; 5 Department of Hematology, Oncology and Palliative Care, Klinikum Magdeburg, Germany; 6 Department Internal Medicine, Clinic III – Hematology, Oncology and Palliative Care, Rostock University Medical Center, Rostock, Germany; 7 Onkologische Gemeinschaftspraxis, Dresden, Germany; 8 Department of Hematology and Oncology, Charité University Hospital Berlin, Germany; current address: Medical Department Oncology, Lilly GmbH, Bad Homburg, Germany; 9 Department of Medicine III, University Hospital Ludwig Maximilians University, Munich, Germany; 10 Department of Hematology and Medical Oncology, Paracelsus Medical University, Nuermberg, Germany; 11 Department of Palliative Care, Jena University Hospital, Jena, Germany; 12 Outpatient Center for Oncology, Westerstede, Germany; 13 Klinik für Innere Medizin - Hämatologie, Onkologie und Palliativmedizin, Vivantes Klinikum Neukölln, Berlin, Germany; 14 Department of Hematology and Oncology, Klinikum Lippe, Lemgo, Germany; 15 Medicine 1 (Oncology, Hematology, Clinical Immunology, and Rheumatology), Saarland University Medical School, Homburg/Saar, Germany; 16 Medical Department A, Hematology and Oncology, University Medicine of Muenster, Germany; 17 Department of Hematology, Oncology and Rheumatology, University Hospital Heidelberg, Germany; 18 Department of Pathology, Hematopathology Section and Lymph Node Registry Kiel, University Hospital Schleswig-Holstein, Kiel, Germany; 19 Institute of Pathology, Comprehensive Cancer Center Mainfranken, University of Würzburg, Germany

## Abstract

The incidence of aggressive B-cell lymphomas increases with age, but for elderly or frail patients not eligible for doxorubicin-containing treatment standard therapy remains to be defined. In this prospective, multicenter, phase-2 B-R-ENDA trial, we investigated the feasibility, toxicity, and efficacy of 8 cycles rituximab combined with 6 cycles bendamustine (BR) in elderly or frail aggressive B-cell lymphoma patients: 39 patients aged >80 years and 29 patients aged 61–80 years with elevated Cumulative Illness Rating Scalescore >6 were included. Progression-free survival (PFS) and overall survival (OS) at 2 years were 45% (95% confidence interval [CI], 28%-61%) and 46% (28%-63%) for the patients age >80, as well 32% (13%-51%) and 37% (17%-57%) for frail patients age 64–80, respectively. In a preplanned retrospective analysis, we found no significant differences in PFS and OS comparing the outcome of the 39 patients age >80 years with 40 patients aged 76–80 years treated with 6xR-CHOP (cyclophosphamide, doxorubicin, vincristine, prednisolone) and 2 x rituximab in the RICOVER-60 trial (DSHNHL 1999-1, NCT00052936, EU-20243), yet we detected lower rates of infections and treatment-related deaths in the BR-treated patients. We demonstrate that older and frail patients with aggressive B-cell lymphoma who are not able to receive standard CHOP-based therapy can benefit from anthracycline-free therapy as a feasible and effective therapeutic option.

This manuscript is dedicated to the memory of Prof Michael Pfreundschuh, founder and president of the DSHNHL, who contributed to the design of this study and passed away much too early in March, 2018.

## INTRODUCTION

The incidence of aggressive B-cell lymphomas is increasing with age.^1–5^ More than 50% of patients are older than 60 years at the time of initial diagnosis. Patients treated with curative intent receive 6 cycles of immunochemotherapy with rituximab, cyclophosphamide, doxorubicin, vincristine, prednisolone (R-CHOP) as standard of care.^6–8^ Toxicity and treatment-related mortality (TRM) increase with age.^9^ In a prospective multicenter phase-II-trial for patients aged 80 years and older with initial diagnosis of aggressive B-cell lymphoma (DLBCL), a dose-reduced protocol of R-miniCHOP-21 every 3 weeks was feasible with a 2-year overall survival (OS) of 59%, 2-year progression-free survival (PFS) of 47%, and a median OS of 29 months.^10^ Most common grades 3–4 adverse events (AE) were neutropenia (40%), anemia (9%), thrombocytopenia (7%), and infections (27%). Another phase-2 trial combined ofatumumab with mini-CHOP as first-line treatment in elderly DLBCL patients aged ≥80 years and showed 2-year OS of 64.7% with grades 3–4 neutropenia in 21% and febrile neutropenia in 6% of patients.^11^ A phase-2 study with ofatumumab and bendamustine as first-line treatment in 21 elderly DLBCL patients aged ≥70 years showed an overall response rates (ORR) of 90.5% and a median PFS and OS of 8.6 and 12.0 months, respectively. The study was closed due to low enrollment.^12^

For older adults or frail patients not eligible for CHOP(-like) therapy standard treatment has not been defined. Bendamustine has been frequently used, however, data from larger prospective trials have not been published. Horn et al^13^ showed in a retrospective trial with 20 patients that rituximab and bendamustine (BR) as first- or second-line treatment is feasible with ORR of 55% and a median OS of 19.4 months. Walter et al^14^ reported in a retrospective analysis of 13 evaluable elderly DLBCL patients an ORR of 62% with a median OS of 9 months. Weidmann et al^15^ demonstrated in a prospective phase-2 trial of 14 patients with aggressive B-cell lymphoma with a median age of 85 years who received BR as first-line treatment an ORR of 69% and an OS of 7.7 months. Bendamustine was given on 2 consecutive days with a dose of 120 mg/m^2^. Most common grade 3 AE were neutropenia, thrombocytopenia, anemia, decreasing renal function, and fatigue.^15^ Recently data of 2 prospective trials with bendamustine in elderly lymphoma patients have been published. Park et al^16^ described an ORR of 78% and a median OS of 10.2 months in 23 previously untreated patients with DLBCL stage II–IV and a median age of 80 years treated with BR using a bendamustine dose of 120 mg/m^2^ on day 1 and 2 of each cycle. Storti et al^17^ reported a prospective phase-II study with BR as front-line therapy in 49 frail patients with a median age of 81 years who achieved an ORR of 62% and a median OS of 30 months using 90 mg/m^2^ bendamustine on days 1–2. The most common grades 3–4 AE was neutropenia (37.8%), 58% of patients received granulocyte-colony stimulating factor (G-CSF).^17^

In older adults and frail patients, a geriatric assessment is among other reasons recommended to evaluate if he or she is fit enough to receive standard treatment. Assessment tools record the performance status, cognitive ability, nutrition status, comorbidities, and mental health.^1,18–28^

To answer the question whether BR is feasible, safe, and effective as first-line treatment in very old or elderly comorbid patients with aggressive B-cell lymphoma, the DSHNHL (Deutsche Studiengruppe für hochmaligne Non-Hodgkin Lymphome; German Study Group for aggressive Non-Hodgkin Lymphoma, now German Lymphoma Alliance, GLA) initiated a prospective multicenter phase-2 trial “Subcutaneous Rituximab and Intravenous Bendamustine in very Elderly Patients or Elderly Medically Non Fit Patients (‘Slow Go’) with Aggressive CD20-positive B-cell Lymphoma” (B-R-ENDA, DSHNHL 2010-1, EudraCT 2010-024004-98, NCT01686321). Geriatric assessment and quality of life (QoL) evaluation was performed using Cumulative Illness Rating Scale (CIRS)-Score,^20,21^ instrumental activities of daily life (IADL),^23,24^ Geriatric Screening Scale G8^27^ and European Organization for Research and Treatment of Cancer (EORTC) QLQ-C30 questionnaire (version 3.0, 1995).

Here we present final results.

## MATERIALS AND METHODS

### Study design

**Figure 1.**
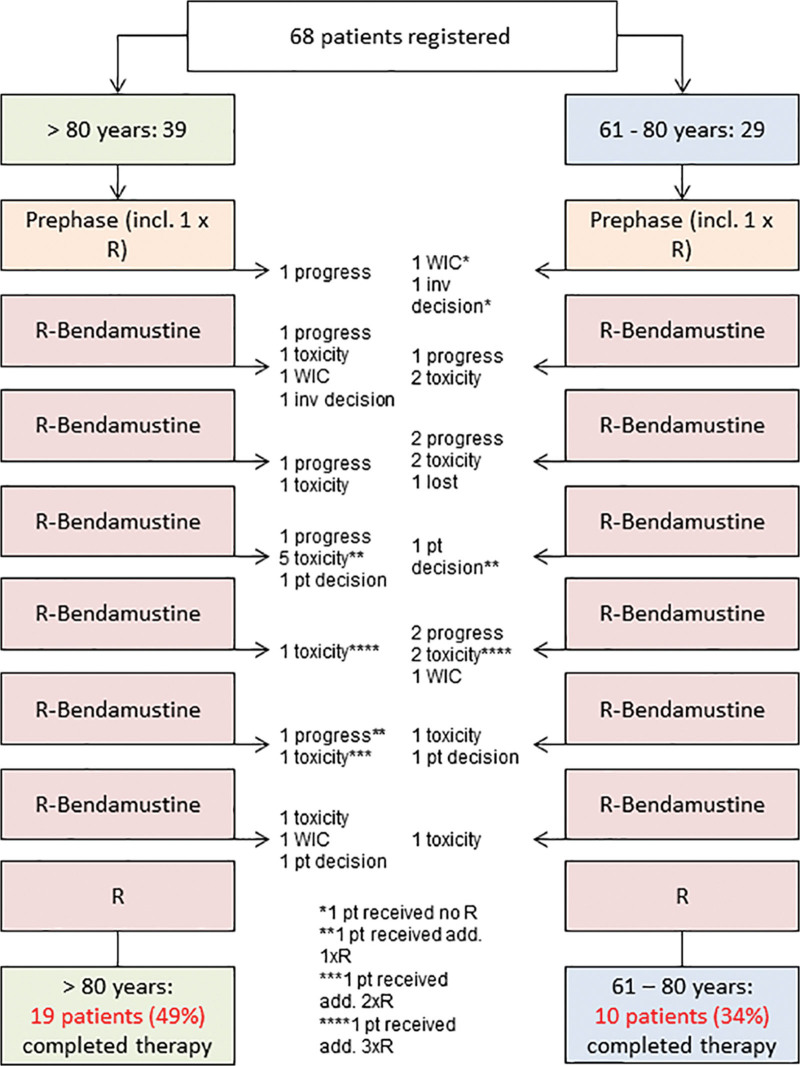
**Consort diagram.** add. = additional; inv = investigator; pt = patient; R = rituximab; WIC = withdraw of informed consent.

The B-R-ENDA trial was an open-label, multicenter, prospective, nonrandomized phase-2 trial including patients aged >80 years or 61–80 years with elevated CIRS score >6 and with histologically confirmed CD20+ aggressive B-cell lymphoma of any Ann-Arbor stage, any international prognostic index (IPI) score^29,30^ and Eastern Cooperative Oncology Group performance score (ECOG PS) <4 determined during prephase treatment, not qualifying for CHOP-like therapy according to exclusion criteria and physician’s opinion. Inclusion and exclusion criteria are given in Suppl. Table S1; consort diagram and flow chart are shown in Figure 1 and Suppl. Figure S1.

Patients received a prephase treatment of prednisolone 100 mg orally on day −7 to −1, followed by rituximab 375 mg/m² intravenously (i.v.) on day −3. The trial treatment consisted of 7 further cycles of rituximab (375 mg/m² i.v. or 1400 mg subcutaneously [s.c.]) on day 1 every 3 weeks and 6 cycles of bendamustine 90 mg/m² i.v. on day 1 and 2 every 3 weeks. During the run-in-phase, 20 patients received rituximab 375 mg/m² i.v. After safety analysis by the Data Safety Monitoring Board, allpatients received an absolute dose of 1400 mg rituximab s.c. G-CSF was recommended following guidelines of the American Society of Clinical Oncology (ASCO) and European Society for Medical Oncology (ESMO) in cases of prolonged neutropenia or infections, a prophylaxis against pneumocystis carinii pneumonia was recommended within the protocol. An interim restaging was performed after 3 cycles (RE1: restaging-1 after 3 cycles). In case of progressive disease (PD) at that time point study treatment ended. Response evaluation was done by computed tomography (CT). In case of complete response (CR), partial response (PR) or stable disease (SD) patients continued therapy. In case of PR or SD in the final restaging (RE2) after 6 cycles of BR treatment, radiation therapy with 39.6 Gray to residual lesions of initial bulky disease (≥7.5 cm lymphoma mass) was performed. These patients received a further restaging after the end of radiotherapy (restaging RE3). Follow-up (FU) observation (3-monthly in years 1 and 2, 6-monthly afterward) within the study ended for all patients 2 years after the end of therapy of the last patient enrolled in the study. The FU period was 2 years minimum and 4.5 years at maximum. The geriatric assessment included 4 elements: CIRS Score,^20,21^ social situation, IADL,^23,24^ G8 (geriatric assessment screening tool).^27^ For QoL evaluation, the EORTC QLQ-C30 questionnaire (version 3.0, 1995) was used.

This study was approved by central and all local ethics committees. All persons participating in the conduct of the trial committed themselves to observe the Declaration of Helsinki and all its revisions and amendments (incl. the Edinburgh Amendment from October 2000), as well as all pertinent national laws and the ICH guidelines for Good Clinical Practice. All patients gave their written informed consent before enrollment.

### Statistical analysis

The aim of the B-R-ENDA trial was to investigate the feasibility, toxicity, and efficacy of BR. The planned sample size of 50 patients in the age group >80 years (target cohort) allowed to establish a robust Kaplan-Meier estimator for the 2-year PFS rate between 30% and 50% with a 95% CI of about ±14% and ±15%, respectively. Patients in the age group ≤80 years represented the exploratory group. Thirty-nine finally included patients aged >80 years enabled the 95% CI of 2-year PFS rate to be estimated with a precision of approximately 15% and 17%. Further primary endpoints for feasibility and toxicity included the rate of treatment-related deaths, AEs, and protocol adherence. Secondary endpoints included CR rate, PR rate, rate of primary progression, relapse rate, OS, EFS, as well as geriatric assessment and QoL evaluation. Curves of duration and absolute dose of bendamustine were estimated according to the Kaplan-Meier technique, and patients with early termination due to insufficient response were censored.^31^ EFS was calculated as time from start of treatment to disease progression, start of salvage treatment, start of any additional, unplanned treatment, SD or unknown response, relapse, or death from any cause. PFS was defined as time from start of treatment to progression, relapse, or death from any cause. OS was defined as time from start of treatment to death from any cause. Patients with no event reported at the time of analysis were censored at the most recent assessment date. Kaplan-Meier curves were presented and Kaplan-Meier estimates at 2 years, with 95% CIs, were calculated for EFS, PFS, and OS. Scoring of the QLQ-C30 was performed according to MANUAL FOR THE USE OF EORTC MEASURES IN DAILY CLINICAL PRACTICE,^32^ with the means of the raw scores for the domains being transformed to lie between 0 and 100. G8, IADL, and QLQ-C30 were descriptively analyzed using mean and range. In a preplanned analysis, the EFS, PFS, and OS of patients from B-R-ENDA trial aged >80 years treated with BR were compared retrospectively with patients from the RICOVER-60 trial (six versus eight cycles of bi-weekly CHOP-14 with or without rituximab in elderly patients with aggressive CD20+ B-cell Lymphomas: A randomised controlled trial [DSHNHL 1999-1, NCT00052936, EU-20243]) aged 76–80 years^6^ treated with 6 cycles of CHOP-14 and 8 applications of rituximab using the log-rank test; Kaplan-Meier curves are presented.^33^ A Cox multivariable regression model was used to test whether therapeutic effects emerging from univariate analyses remained stable after adjustment for the factors of the IPI (ie, age >60 years, lactate dehydrogenase [LDH] > normal, ECOG PS >1, stage III/IV, and extralymphatic involvement >1).^29^ Estimates are given as hazard ratios with 95% CI and corresponding *P* values. Subgroup analyses were done according to the number of IPI factors (1, 2 vs 3–5), according to CIRS (≤6 vs >6), G8 (≥14 vs <14), IADL (=8 vs <8), and Quality of Life (EORTC) (≥50 vs <50, Global Score). Patient characteristics were analyzed by use of χ^2^ test and, if necessary, by Fisher exact test. Significance level was 0.050. Statistical analyses were done with IBM SPSS 25 and 26 software.

### Data sharing statement

Individual patient information underlying the data presented in this article can be shared after deidentification. Researchers have to provide a proposal for an approval from an independent review committee to access these data. The protocol and informed consent forms will be available on request beginning 3 months and ending 5 years after publication. Requests can be addressed to the corresponding author.

## RESULTS

### Patients

Between July 2012 and February 2016, 68 patients from 24 German centers were included in the trial (Table 1): 39 patients aged >80 years, and 29 patients aged 61–80 years. The geriatric assessments by CIRS/G8/IADL were performed by physicians, so they were available at initial staging for all patients. QoL was evaluated by paper-based questionnaires according to EORTC QLQ-C30 questionnaire (version 3.0, 1995)^32^ and was available at staging for 46 of 68 patients. In patients >80 years, 72% were female, LDH was elevated in 67% of patients, 23% of patients had an ECOG >1, 51% of patients were at an advanced stage III/IV according to Ann-Arbor classification, 54% showed extralymphatic involvement, 10% in more than one localization resulting in higher IPI scores 3–5 in 46% of patients. The median CIRS score for patients >80 years was 7 (range 1–17), 69% of patients had a G8 score <14, 43% had an IADL score of 8, and the median score of Quality of Life (EORTC) was 58 (8–100). Patients aged 61–80 years were medically nonfit according to the protocol criteria. Patients >80 years were fitter than patients aged 61–80 years at initial screening. The rates of elevated LDH (76%), ECOG >1 (52%), and stage III/IV (66%) were higher for patients aged 61–80 years and the values for geriatric assessment were poorer. The median CIRS score was 10 (2–22) including 2 protocol violations regarding CIRS >6, 93% of patients had a G8 score <14, 14% had an IADL score of 8, and the median of Quality of Life (EORTC) was 33 (0–83). At initial staging, 17 patients (25%) lived at home alone, 47 (69%) lived at home with someone else, and 4 patients (6%) stayed in institutional care. Central reference pathology according to protocol was available in 91% of patients (62/68) using World Health Organization (WHO) classification of 2008.^34^ Histologic subtypes were diffuse large B-cell lymphoma (DLBCL) not otherwise specified, follicular lymphoma grade IIIb, and other in decreasing frequency (Table 1). For 1 patient >80 years, the diagnosis changed to chronic lymphatic leukemia. Two further patients violated the inclusion criteria—1 patient aged 61–80 years received prior chemotherapy or radiotherapy and another patient >80 years had a concomitant solid tumor disease and/or tumor disease in the past 5 years (Table 1).

**Table 1:** Demographics and Disease Characteristics for Patients at the Time of Initial Staging

	>80 y (n = 39)	61–80 y (n = 29)	Total (n = 68)
Male	11 (28%)	11 (38%)	22 (32%)
Female	28 (72%)	18 (62%)	46 (68%)
Age, median (range)	84 (81–95)	77 (64–80)	81 (64–95)
Age groups			
61–75 y	-	8 (28%)	8 (12%)
76–80 y	-	21 (72%)	21 (31%)
81–85 y	25 (64%)	-	25 (37%)
>85 y	14 (36%)	-	14 (21%)
LDH > normal	26 (67%)	22 (76%)	48 (71%)
ECOG > 1	9 (23%)	15 (52%)	24 (35%)
Stage III/IV	20 (51%)	19 (66%)	39 (57%)
Extralymphathic involvement^a^	21 (54%)	19 (66%)	40 (59%)
Extralymphathic involvement >1	4 (10%)	7 (24%)	11 (16%)
IPI			
1	6 (15%)	2 (7%)	8 (12%)
2	15 (38%)	5 (17%)	20 (29%)
3	11 (28%)	12 (41%)	23 (34%)
4, 5	7 (18%)	10 (34%)	17 (25%)
Bulky disease	8 (21%)	11 (38%)	19 (28%)
B symptoms	10 (26%)	14 (48%)	24 (35%)
Bone marrow involvement	0 (0%)	2 (7%)	2 (3%)
Reference pathological diagnosis			
Reviewed	36	26	62
Diffuse large B-cell lymphoma (DLBCL)			
Not otherwise specified	28 (78%)	23 (88%)	51 (82%)
Rare morphologic variants	0 (0%)	1 (4%)	1 (2%)
T-cell/histiocyte-rich B-cell lymphoma	0 (0%)	1 (4%)	1 (2%)
Primary cutaneous DLBCL, leg type	1 (3%)	1 (4%)	2 (3%)
EBV-positive DLBCL of the elderly	1 (3%)	0 (0%)	1 (2%)
Primary mediastinal (thymic) large B-cell lymphoma	1 (3%)	0 (0%)	1 (2%)
Follicular lymphoma grade IIIb	3 (8%)	0 (0%)	3 (5%)
ALK-positive large B-cell lymphoma	1 (3%)	0 (0%)	1 (2%)
No lymphoma (CLL)	1 (3%)	0 (0%)	1 (2%)
CIRS, median (range)	7 (1–17)	10 (2–22)	8 (1–22)
CIRS			
≤6	17 (44%)	2^b^ (7%)	19 (28%)
>6	22 (56%)	27 (93%)	49 (72%)
Geriatric assessment G8, median (range)	12 (4–16)	11 (6–15)	11 (4–16)
G8			
≥14	12 (31%)	2 (7%)	14 (21%)
<14	27 (69%)	27 (93%)	54 (79%)
IADL^c^			
=8	16 (43%)	4 (14%)	20 (30%)
<8	21 (57%)	25 (86%)	46 (70%)
EORTC^d^			
Quality of life^e^, median (range)	58 (8–100)	33 (0–83)	50 (0–100)
Social situation			
At home alone	12 (31%)	5 (17%)	17 (25%)
At home with someone	25 (64%)	22 (76%)	47 (69%)
In institutional care	2 (5%)	2 (7%)	4 (6%)

Data are expressed as number of patients (percentage of total group).

aBone marrow involvement is counted as extralymphatic involvement, spleen and Waldeyer Ring are counted as lymphatic involvement.

bTwo patients with violation of inclusion criterion.

cTwo missing values for patients >80 y.

d22 (10/12) missing questionnaires.

eOne missing value for patients >80 y.

Bulky disease = .7.5 cm lymphoma mass; CIRS = Cumulative Illness Rating Scale; CLL = chronic lymphatic leukemia; ECOG = Eastern Cooperative Oncology Group performance score; EBV = Epstein-Barr virus; EORTC = European Organization for Research and Treatment of Cancer; G8 = Geriatric Screening Scale G827; IADL = instrumental activities of daily life; IPI = international prognostic index; LDH = lactate dehydrogenase.

### Adherence to protocol

All patients received prephase treatment. Fifty-six percent of patients aged >80 years (22/39) and 38% (11/29) of patients aged 61–80 years completed 6 cycles of treatment per protocol (Figure 1). Early termination was due to PD (>80 years: n = 5; 61–80 years: n = 5), toxicity (n = 9; n = 7), withdrawal of consent (n = 1; n = 2), investigator’s decision (n = 1; n = 1), lost to FU (n = 0; n = 1), and patient’s wish (n = 1; n = 2). Four further patients did not receive the eighth rituximab application due to toxicity (n = 1; n = 1), withdrawal of consent (n = 1; n = 0), or patient’s wish (n = 1; n = 0). The duration of therapy according to study protocol ranged between 20 and 22 weeks for patients receiving immunochemotherapy only and 24–26 weeks for those with additional radiotherapy. For both age groups, the median total duration of bendamustine is as planned, but treatment delays increased with the duration of therapy (Suppl. Figure S2A). The median absolute dose of bendamustine was slightly smaller than the planned dose of 1080 mg/m² (>80 years: 1026 mg/m²; 61–80 years: 1021 mg/m²) due to the high number of patients with early termination of the treatment (Suppl. Figure S2B). Interestingly, neither the total duration nor the absolute dose for bendamustine or rituximab differs among the 2 age groups. Radiotherapy was planned for all patients with bulky lesions who do not show a CR after 6 cycles of bendamustine and a total of 8 applications of rituximab. Three patients received radiotherapy: 2 patients with bulky disease and either SD or partial remission after immunochemotherapy, and another patient without bulky disease and CR after immunochemotherapy for extranodal involvement. Only 2 patients (1 PR, 1 SD), both aged <80 years, did not receive consolidation radiation therapy due to physician’s choice and patient wish.

### Safety

AE and deaths occurring during the study were continuously monitored by the trial office and documented in an annual safety report. The benefit–risk evaluation was considered favorable. AE were classified according to National Cancer Institute Common Terminology Criteria for Adverse Events (NCI-CTCAE V4.03). In total, 849 AE and 118 serious AE (SAE) of any grade were documented (Table 2): most common AE of any grade were hematological toxicities (11%), gastrointestinal disorders (18%), general disorders (16%), and infections (10%). For grades 3–5 AE (n = 181) hematological toxicities (25%), infections (15%), metabolism and nutrition (13%), and gastrointestinal disorders (8%) were the most frequent. The rate of patients affected aged 61–80 years compared to patients >80 years was higher for infections (38% vs 23%), gastrointestinal disorders (24% vs 15%), and respiratory disorders (14% vs 8%). In Table 2, all AE are listed, which occurred in at least 5% of patients within the 2 cohorts. Secondary neoplasia was detected in 4 patients (6%): 2 carcinoma (bronchial and renal cell carcinoma) and 1 acute myeloid leukemia and 1 myelodysplastic syndrome, both with a median of 19.5 months after end of treatment (Suppl. Table S2). One Suspected Unexpected Serious Adverse Reaction (SUSAR) was reported that has emerged from an event of pulmonary embolism possibly related to the application of bendamustine. Patient information was changed; the risk profile of the whole trial had not changed. All nonlymphoma-related deaths during therapy until 60 days after treatment termination were counted as study treatment-related deaths. There were 5 treatment-related deaths within patients >80 years (13%) and 5 treatment-related deaths within patients aged 61–80 years (17%), most of them infections (70%), followed by cardiovascular events (30%).

**Table 2:** AE of Any Grade (≥5% of All AEs in at Least One of the Cohorts, Listed), AEs Grades 3–5 and SAEs According to NCI-CTCAE V4.03

	>80 y (n = 39)					61–80 y (n = 29)					Total (n = 68)				
	Adverse Events			Serious Adverse Events		Adverse Events			Serious Adverse Events		Adverse Events			Serious Adverse Events	
	Any Grade (n = 454)	Grades 3–5 (n = 102)		Any Grade (n = 56)	Grades 3–5 (n = 38)	Any Grade (n = 395)	Grades 3–5 (n = 79)		Any Grade (n = 62)	Grades 3–5 (n = 39)	Any Grade (n = 849)	Grades 3–5 (n = 181)		Any Grade (n = 118)	Grades 3–5 (n = 77)
Event	n (%)	n (%)	Affected Patients n (%)	n (%)	n (%)	n (%)	n (%)	Affected Patients n (%)	n (%)	n (%)	n (%)	n (%)	Affected Patients n (%)	n (%)	n (%)
Blood and lymphatic system disorders	58 (13)	28 (28)	17 (44)	4 (7)	4 (11)	33 (8)	17 (22)	10 (35)	2 (3)	2 (5)	91 (11)	45 (25)	27 (40)	6 (5)	6 (8)
Cardiac disorders	16 (4)	6 (6)	5 (13)	4 (7)	4 (11)	18 (5)	5 (6)	4 (14)	6 (10)	5 (13)	34 (4)	11 (6)	9 (13)	10 (8)	9 (12)
Gastrointestinal disorders	75 (17)	7 (7)	6 (15)	8 (14)	4 (11)	75 (19)	8 (10)	7 (24)	8 (13)	4 (10)	150 (18)	15 (8)	13 (19)	16 (14)	8 (10)
General disorders and administration site conditions	77 (17)	8 (8)	4 (10)	7 (12)	3 (8)	60 (15)	4 (5)	4 (14)	7 (11)	3 (8)	137 (16)	12 (7)	8 (12)	14 (12)	6 (8)
Infections and infestations	39 (9)	11 (11)	9 (23)	12 (21)	10 (26)	42 (11)	16 (20)	11 (38)	18 (29)	12 (31)	81 (10)	27 (15)	20 (29)	30 (25)	22 (29)
Metabolism and nutrition disorders	37 (8)	12 (12)	10 (26)	1 (2)	1 (3)	42 (11)	11 (14)	9 (31)	4 (6)	2 (5)	79 (9)	23 (13)	19 (28)	5 (4)	3 (4)
Nervous system disorders	19 (4)	4 (4)	3 (8)	3 (5)	2 (5)	18 (5)	2 (3)	2 (7)	2 (3)	2 (5)	37 (4)	6 (3)	7 (7)	5 (4)	4 (5)
Respiratory, thoracic and mediastinal disorders	26 (6)	3 (3)	3 (8)	4 (7)	2 (5)	24 (6)	4 (5)	4 (14)	3 (5)	2 (5)	50 (6)	7 (4)	7 (10)	7 (6)	4 (5)

AE = adverse events; NCI = National Cancer Institute (US National Institute of Health); CTCAE = common toxicity criteria for adverse events; SAE = serious AE; V = version.

### Efficacy

The planned sample size was addressed to patients aged >80 years: 20 of 39 patients >80 years had an overall response (51%; 95% CI: 35%-68%). CR was achieved in 18 of 39 (46%; 30%–63%) and PR in 2 of 39 (5%) patients. There were 2 (5%) patients with SD, 5 (13%) with PD, and 7 (18%) patients with unknown response (Table 3). Relapse after CR occurred in 2 of 18 patients (11%).

**Table 3:** Treatment Response

	>80 y (n = 39)	61–80 y (n = 29)	Total (n = 68)
**Treatment response n (%**)			
CR (95% CI)	18 (46) (30-63)	3 (10) (2-27)	21 (31) (20-43)
CR and additional treatment	0 (0)	2^a^ (7)	2 (3)
PR (95% CI)	2 (5) (1-17)	5 (17) (6-36)	7 (10) (4-20)
SD	2 (5)	1 (3)	3 (4)
PD (95% CI)	5 (13) (4-27)	9 (31) (15-51)	14 (21) (12-32)
Unknown	7 (18)	4 (14)	11 (16)
Study treatment-related death	5 (13)	5 (17)	10 (15)
Relapse after CR (95% CI)	2/18 (11) (1-35)	0/3 (0) (0-71)	2/21 (10) (1-30)
**EFS, PFS, OS rates with 95% CI**			
2-y EFS	33 (18-48)	10 (0-21)	23 (13-33)
2-y PFS	45 (28-61)	32 (13-51)	40 (27-52)
2-y OS	46 (28-63)	37 (17-57)	42 (29-55)
**PFS event n (%**)	21/39 (54)	18/29 (62)	39/68 (57)
PD	5 (24)	9 (50)	14 (36)
Progression after PR, SD, unknown	3 (14)	1 (6)	4 (10)
Relapse after CR/CR and additional treatment	2 (10)	1 (6)	3 (8)
Death as earliest event	11 (52)	7 (39)	18 (46)
**Cause of death n (%**)	19/39 (49)	16/29 (55)	35/68 (51)
Lymphoma related	6 (32)	7 (44)	13 (37)
Study treatment related	5 (26)	5 (31)	10 (29)
Secondary neoplasia	0 (0)	1 (6)	1 (3)
Concomitant disease	1 (5)	1 (6)	2 (6)
Unknown	7 (37)	2 (12)	9 (26)

aOne patient received additional radiotherapy, 1 patient received additional rituximab.

CI = confidence interval; CR = complete remission; EFS = event-free survival; OS = overall survival; PD = progressive disease; PFS = progression-free survival; PR = partial remission; SD = stable disease.

**Figure 2.**
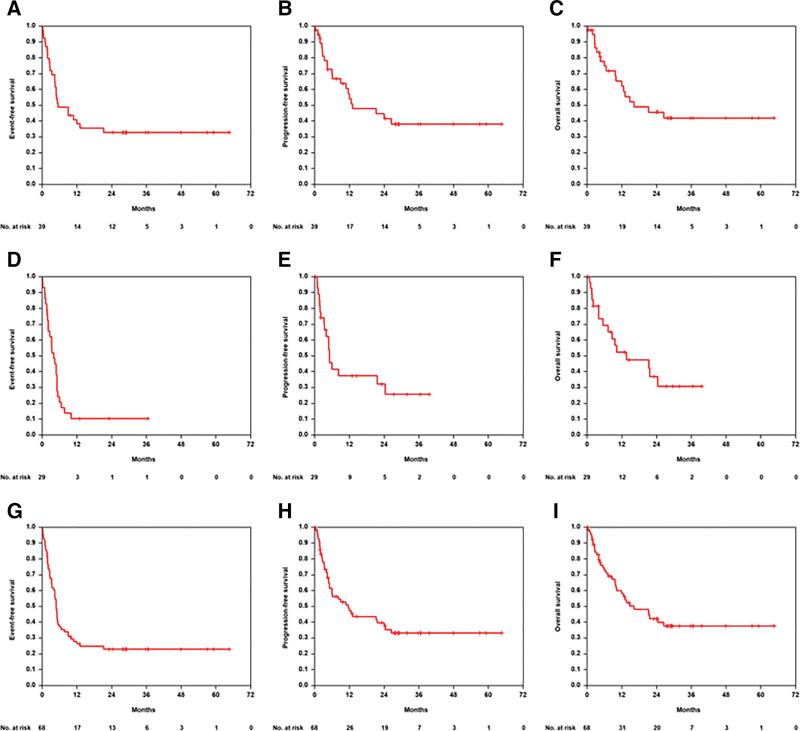
**EFS, PFS and OS in B-R-ENDA treated patients.** Event-free-survival (A, D, G), progression-free survival (B, E, H), and overall survival (C, F, I) in 39 patients aged >80 y (A–C), 29 patients aged 61–80 y (D–F), and for the 68 patients of the full analysis set (G–I).

With a median observation time for OS of 29 months (range 0–65) for patients aged >80 years median EFS, PFS, and OS were 5, 13, and 16 months, respectively (Figure 2). Two-year EFS, 2-year PFS, and 2-year OS were 33% (95% CI, 18%-48%), 45% (28%-61%), 46% (28%-63%), respectively. The EFS, PFS, and OS curves show better (but not significant) outcome for patients with IPI 1, 2 versus IPI 3–5 (Suppl. Figure S3). There were no significant differences for EFS, PFS, and OS between male and female patients (data not shown). For EFS, there was no difference between the group with lower (≤6, n = 17) and higher (>6, n = 22) CIRS score (*P* = 0.399) in patients >80 years, but for PFS (*P* = 0.165) and OS (*P* = 0.049) patients with CIRS >6 had improved outcome (Suppl. Figure S4), possibly due to an imbalance of risk factors in this small subgroup. There were no significant differences in EFS, PFS, and OS according to G8 ≥14 (n = 12) versus G8 <14 (n = 27) in patients >80 years with a trend toward improved OS in patients with higher G8 scores (*P* = 0.068, Suppl. Figure S5). According to IADL, there was a significant better PFS (*P* = 0.038) and OS (*P* = 0.035) in patients aged >80 years and higher IADL score (Suppl. Figure S6). EFS, PFS, and OS according to QoL (EORTC) differed not significantly in patients aged >80 years between QoL Scores ≥50 (n = 19) versus QoL score <50 (n = 9, Suppl. Figure S7). In a multivariable analysis adjusted for IPI factors elevated LDH, ECOG >1 and >1 extralymphatic involvement constituted the most relevant risk factors for poor OS (Table 4). In total, 19 of 39 patients >80 years (49%) died: 6 of 19 (32%) tumor related, 5 of 19 (26%) treatment related, 1 of 19 (5%) due to concomitant disease and in 7 of 19 patients (37%), the cause of death remains unknown (Table 3).

**Table 4:** Multivariable Analysis for EFS, PFS, and OS for Patients >80 y

	EFS HR (95% CI)	*P*	PFS HR (95% CI)	*P*	OS HR (95% CI)	*P*
LDH > normal	2.6 (1.0-6.5)	0.049	4.2 (1.2-14.8)	0.024	4.3 (1.2-15.8)	0.026
ECOG > 1	2.1 (0.8-5.6)	0.116	2.4 (0.8-7.2)	0.136	3.8 (1.2-12.2)	0.028
Extralymphathic involvement > 1	3.2 (0.9-11.3)	0.075	3.5 (0.9-13.1)	0.067	6.4 (1.5-27.4)	0.013
Stage III/IV	0.9 (0.4-2.1)	0.759	0.7 (0.2-2.0)	0.492	0.4 (0.1-1.4)	0.147

CI = confidence interval; ECOG = Eastern Cooperative Oncology Group performance score; EFS = event-free survival; HR = hazard ratio; LDH = lactate dehydrogenase; OS = overall survival; PFS = progression-free survival.

**Figure 3.**
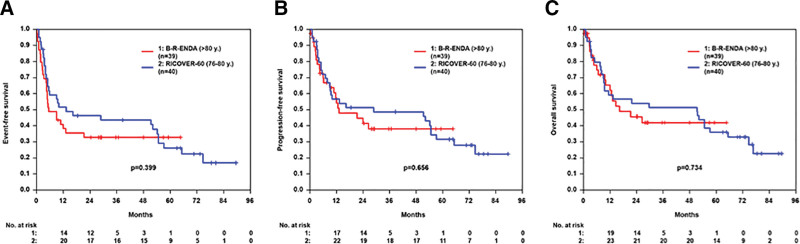
**EFS, PFS and OS in elderly B-R-ENDA and RICOVER-60 patients.** Event-free-survival (A), progression-free survival (B), and overall survival (C) of B-R-ENDA patients aged >80 y (n = 39) and RICOVER-60 patients (6xCHOP 14 + 8xR) aged 76–80 y (n = 40).

In a preplanned additional analysis, we compared the outcome of the 39 patients >80 years with 40 patients aged 76–80 years treated within the RICOVER-60 trial with 6xCHOP and 8 applications rituximab. There were no significant differences according to patient characteristic, except sex (52% male patients within RICOVER-60 vs 28% within B-R-ENDA, *P* = 0.028), bulky disease (42% within RICOVER-60 vs 21% within B-R-ENDA, *P* = 0.036), and age (Suppl. Table S3). Twenty-three percent of patients >80 years treated within the B-R-ENDA trial terminated the study early due to toxicity compared to 33% within the RICOVER-60 trial. The number of patients with an infection grades 3–5 was 23% within the B-R-ENDA trial (patients >80 years) and 44% within RICOVER-60 (patients aged 76–80 years). The TRM was 13% within the B-R-ENDA trial and 20% within RICOVER-60. EFS, PFS, and OS were not significantly different between these 2 cohorts for all patients (Figure 3) and for patients with IPI 1, 2, or 3–5 (Suppl. Figure S8). Two-year EFS, 2-year PFS, and 2-year OS for the 40 RICOVER-60 patients were 46% (95% CI, 31%-62%), 51% (36%-67%), 54% (38%-70%), respectively.

As expected based on the inclusion criteria for this age group, the outcome of patients aged 61–80 years within the B-R-ENDA trial (n = 29) was inferior in comparison to the patients aged >80 years. Three patients achieved CR and 5 PR, the ORR was 28% (13%–47%). Two further patients achieved a CR, but the investigators decided to treat the patient additionally with radiotherapy or rituximab. There was 1 (3%) patient with SD, 9 (31%) patients with PD, and 4 (14%) patients with unknown response (Table 3). No relapse was reported. Five patients (17%) deceased during treatment due to toxicity. With a median observation time of 27 months for OS (range 0–40) for patients aged 61–80 years median EFS, PFS, and OS were 4, 5, and 14 months, respectively (Figure 2). Two-year EFS, PFS, and OS were 10% (0%–21%), 32% (13%–51%), 37% (17%–57%), respectively. In total, 16 of 29 patients aged 61–80 years (55%) have died: 7 of 16 (44%) lymphoma related, 5 of 16 (31%) treatment related, 1 of 16 (6%) due to secondary neoplasia, 1 of 16 (6%) due to concomitant disease, and in 2 of 16 patients (12%), the cause of death remains unknown (Table 3).

## DISCUSSION

Although the incidence of aggressive B-cell lymphoma is increasing with age, patients older than 80 years or frail patients are excluded from most clinical trials. We sought to investigate the feasibility, toxicity, and efficacy of BR in such patients. Due to slow recruitment, the B-R-ENDA trial had to be terminated before the planned target was reached once more illustrating the difficulty of performing trials in this frail patient population.^12,15^ Nevertheless, this study represents the largest prospective cohort of elderly and frail patients with aggressive B-cell lymphoma and BR as first-line treatment reported so far.

According to protocol, patients >80 years were fitter than younger patients aged 61–80 years who also presented with higher LDH rates, more advanced stage and poorer performance status, higher CIRS and lower QoL scores. All patients received prephase treatment, but only half of the patients completed treatment per protocol, most of them (56%) were >80 years. Early termination of therapy was more frequent in patients ≤80 years, the major reasons being toxicity and PD.

Geriatric assessment was performed at initial screening and during FU and will be presented in further analyses in detail. Only for IADL screening the curves separated significantly for improved PFS and OS in patients aged > 80 years and higher IADL scores.

Only 2 patients with initial bulky disease and PR and SD as best response after immunochemotherapy did not receive consolidative radiation therapy, as it is recommended in Germany, based on physician’s choice and patient’s wish. Today, a [18F]fluorodeoxyglucose–positron emission tomography (PET) CT would be recommended to decide about consolidation radiation therapy in bulky disease DLBCL patients.^35^

Blood and lymphatic system disorders were the most common grades 3–5 AE (25%, 45/181) followed by infections (15%, 27/181), 38% of patients aged ≤80 years and 23% >80 years developed grades 3–5 infections. In total, 29% (22/77, Table 2) of all grades 3–5 SAE in B-R-ENDA treated elderly and frail patients were infections. In comparison, 27% (19/70) of all grades 3–5 SAE in R-miniCHOP treated elderly patients described by Peyrade et al^10^ were infections and infestations. We could demonstrate that there were 23% of B-R-ENDA treated patients aged >80 years with severe infections (grades 3–5) compared with 44% of RICOVER-60 treated patients aged 76–80 years. In both trials, the use of G-CSF was recommended according to ASCO/ESMO guidelines. In B-R-ENDA–treated patients, G-CSF was administered in 38% of patients aged >80 years and 31% of patients aged 61–80 years. Storti et al^17^ reported on only 4.4% grades 3–4 AE with infections in an elderly patient cohort comparable to our B-R-ENDA trial. Out of these patients 58% received G-CSF.^17^

The assessment of TRM was performed very conservatively, since all events until 60 days after treatment termination were counted as treatment-related. In total, 5 patients aged >80 years (13%) and 5 patients aged 61–80 years (17%) died of treatment-related causes. This is a higher rate than described by Storti et al^17^ for elderly frail patients treated with front-line BR (6%) or by Peyrade et al^10^ for R-miniCHOP–treated elderly patients (12/150 deaths, 8%). For patients treated within the RICOVER-60 trial, TRM was higher (20%) in patients aged 76 to 80 years.^9^

In former studies,^30^ low-risk-IPI patients showed significantly better EFS, PFS, and OS. In our cohort, multivariable analyses adjusted for IPI factors showed that even in older adults aged >80 years elevated LDH, ECOG >1 and more than 1 extralymphatic involvement remain the most relevant risk factors for poor outcome in contrast to stage. We could demonstrate that IPI allows a good risk stratification of lymphoma patients, while the CIRS score was not an appropriate tool for differentiation of patients at risk. Thus, the predictive power of the established IPI factors stays the same even in old patients.

For all B-R-ENDA trial patients ORR was 41%. Patients aged >80 years, who completed therapy more often than patients aged 61–80 years with higher CIRS score, showed an ORR of 51%. Prior trials using BR as first-line treatment in elderly patients with aggressive lymphoma described slightly higher ORR of 62%,^17^ 69%,^15^ and 78%.^16^

Age alone does not seem to be the most important factor. Our data show, that older but fitter patients with a lower CIRS score had higher adherence to therapy, achieved higher response rates and less relapses. The 2-year PFS of 45% in B-R-ENDA–treated patients aged >80 years was comparable to 47% 2-year PFS in R-miniCHOP–treated elderly patients described by Peyrade et al.^10^ With a median OS of 16 months, the combination of BR as first-line treatment seems to be less effective than rituximab combined with miniCHOP with a median OS of 29 months^10^ but associated with a comparable rate of infections. The cohort was too small for further subgroup analyses except IPI factors. BR should not replace R-miniCHOP regimes, but our data show that older or frail DLBCL patients not able to receive standard CHOP-based therapy can benefit from that anthracycline-free therapeutic option.

At the time of planning the B-R-ENDA trial, the results of the R-miniCHOP trial were not yet available. Meanwhile, also miniCHOP together with ofatumumab^11^ or lenalidomide^36^ and new antibody-drug conjugates have shown promising results for elderly lymphoma patients either as monotherapy^37^ or in combination with BR^38^ or modified R-CHOP^39^ regimen. Again, infections and infestations remain a serious challenge for patients and physicians.^36–39^ Polatuzumab vedodtin combined with BR^40^ might be an interesting option to study for elderly and frail DLBCL patients even for up-front treatment. Further prospective and randomized trials are needed to identify new treatment approaches to better manage the dilemma between increased toxicity and reduced efficiency in elderly and frail DLBCL patients. For future trials with old and comorbid patients, there should be clear recommendations for optimal supportive care including antiinfectious prophylaxis, the use of G-CSF, and PET-CT driven decision-making.^35^

## ACKNOWLEDGMENTS

The authors would like to thank all patients and their families for participating in this trial and all colleagues at each clinical trial site for supporting this study. All authors like to thank IOMEDICO AG, Freiburg, for trial organization, as well as Elke Stitz for trial office management at the University Medicine of Goettingen, and Beate Mann and Katja Rillich for technical assistance at the University of Leipzig.

## AUTHOR CONTRIBUTIONS

LT, MZ, FZ, FB, UW did study conception and design. All the authors collected and assembled the data; provision of study material and patient care. BA, MZ, FB, FZ, LT did data analysis and interpretation. FB, BA, MZ, FZ, LT drafted or revised the manuscript. FB, FZ, MZ, AV, CK, GPK, AK, MD, AB, UW, DR, MdW, FH, VP, NS, MWH, WK, AR, GW, BA, LT reviewed and approved the final version of the manuscript. All contributing centers are listed in Suppl. Table S4.

## DISCLOSURES

AV was part of an advisory Board and lectures for Novartis, BMS, Kite/Gilead, Roche and Amgen. GP-K was part of an advisory board for Janssen and Abbvie. AK Employment at Lilly Deutschland GmbH since 2018. MD received Research support (institution) by Abbvie, Bayer, BMS/Celgene, Gilead/Kite, Janssen, Roche, and received speakers honoraria by Amgen, Astra Zeneca, BMS/Celgene, Gilead/Kite, Incyte, Janssen, Novartis, Roche; was part of an scientific advisory board for Astra Zeneca, Beigene, BMS/Celgene, Genmab, Gilead/Kite, Incyte, Janssen, Lilly/Loxo, Morphosys, Novartis, Roche. AB was part of an advisory board for Incyte, received travel support by BMS, Novartis and Roche. VP received travel support by Abbvie, Amgen, BMS, Gilead and Roche. GW was part of an advisory board and performed lectures for Novartis, Kite/Gilead, Roche, Amgen, Takeda, Clinigen. All the other authors have no conflicts of interest to disclose.

## SOURCES OF FUNDING

This trial was supported by Astellas Pharma GmbH, Munich, Germany (trial medication bendamustine) and Roche AG, Basel, Swiss (trial medication rituximab i.v. and s.c.). They provided free trial medication and an unrestricted grant for trial conduct.

## Supplementary Material


