# Supplementary material for: First-line Treatment With Bendamustine and Rituximab for Old and Frail Patients With Aggressive Lymphoma: Results of the B-R-ENDA Trial
Source: Hemasphere. 2022 Dec 1;6(12):e808. doi: 10.1097/HS9.0000000000000808 (PMC9722574; doi:10.1097/HS9.0000000000000808)

**Supplement**

**Supplemental Table 1: Inclusion criteria and exclusion criteria.**

**Supplemental Table 2: Secondary neoplasia (n=4).**

**Supplemental Table 3: Characteristics of B-R-ENDA patients aged > 80 years (n=39) and RICOVER-60 patients aged 76 – 80 years (n=40).**

**Supplemental Table 4: Contributing centers.**

**Supplemental Figure 1: Flow chart.**

**Supplemental Figure 2: Duration and absolute dose of Bendamustine therapy.**

**Supplemental Figure 3: EFS (A), PFS (B) and OS (C) according to IPI 1, 2 (n=21) and 3-5 (n=18) for B-R-ENDA patients > 80 years of age.**

**Supplemental Figure 4: EFS (A), PFS (B) and OS (C) according to CIRS ≤ 6 (n=17) and CIRS > 6 (n=22) for B-R-ENDA patients > 80 years of age.**

**Supplemental Figure 5: EFS (A), PFS (B) and OS (C) according to G8 ≥ 14 (n=12) and G8 < 14 (n=27) for B-R-ENDA patients > 80 years of age.**

**Supplemental Figure 6: EFS (A), PFS (B) and OS (C) according to IADL = 8 (n=16) and IADL < 8 (n=21) for B-R-ENDA patients > 80 years of age (two missing values for IADL).**

**Supplemental Figure 7: EFS (A), PFS (B) and OS (C) according to Quality of Life (EORTC) ≥ 50 (n=19) and Quality of Life (EORTC) < 50 (n=9) for B-R-ENDA patients > 80 years of age.**

**Supplemental Figure 8: Event-free-survival (A, D), progression-free survival (B, E) and overall survival (C, F) of B-R-ENDA patients aged > 80 years (n=39) and RICOVER-60 patients patients (6xCHOP-14+8xR) aged 76 – 80 years (n=40) according to low (A – C) and high (D – F) IPI scores.**

**Supplemental Table 1: Inclusion criteria and exclusion criteria.**

| **Inclusion Criteria** | **Exclusion Criteria** |
| --- | --- |
| 1. Histology: Diagnosis of aggressive CD20+, confirmed by an excisional biopsy of a lymph node or by a sufficiently extensive biopsy of an extranodal involvement if there is no lymph node involvement. It will be possible to treat the following entities in this study as defined by the new WHO classification of 2008:   - B-NHL: - Follicular lymphoma grade IIIb - DLBCL, not otherwise specified (NOS) - common morphologic variants:   centroblastic, immunoblastic, anaplastic   - rare morphologic variants - DLBCL subtypes/entities:   T cell/histiocyte rich large B-cell lymphoma,  primary cutaneous DLBCL, leg type,  EBV-positive DLBCL of the elderly   - DLBCL associated with chronic inflammation - primary mediastinal (thymic) large B-cell lymphoma - intravascular large B-cell lymphoma - ALK-positive large B-cell lymphoma - Plasmoblastic lymphoma - Primary effusion lymphoma - B-cell lymphoma, unclassifiable, with features intermediate between diffuse large B-cell lymphoma and Burkitt lymphoma - B-cell lymphoma, unclassifiable, with features intermediate between diffuse large B-cell lymphoma and Hodgkin lymphoma   2. Stage: Any stages according to Ann Arbor Classification  3. Risk group: All risk groups (IPI 1 to 5)  4. Life expectancy Life expectancy of at least 6 weeks, when lymphoma is treated  5. Age: Age ≥ 81 or Age 61 to 80 and CIRS >6 not qualifying for CHOP-therapy  6. Gender: any  7. Performance status: Performance status ECOG <4. The performance status of each patient is to be assessed at the time of registration which might be after the initiation of pre-phase treatment up to the first rituximab application which, as experience has shown, can result in a significant improvement of the patient´s performance status.  8. Ability to give informed consent  9. Written informed consent of the patient  10. Contract of participation signed by the study center and sponsor | 1. Already initiated lymphoma therapy (except for the prephase treatment until first application of rituximab)  2. Serious accompanying disorder or impaired organ function (except when due to lymphoma involvement), in particular:   - Heart: angina pectoris CCS >2, cardiac failure NYHA >3 - Lungs: the patient is to be excluded if the resultant pulmonary function test shows FEV1<50% or a diffusion capacity <50% of the reference values - Calculated creatinin clearance < 10 ml/min (Cockcroft-Gault) - Liver: total bilirubin > 3 mg/dl - Uncontrollable diabetes mellitus (because of prophase treatment with prednisone!)   3. Platelets <100 000/mm3, leukocytes <2500/mm3 (if not due to lymphoma)  4. Known hypersensitivity to the medications to be used  5. HIV-positivity  6. Acute or chronic active hepatitis  7. Poor patient compliance  8. Simultaneous participation in other treatment studies  9. Prior chemo- or radiotherapy, long-term use of corticosteroids or anti-neoplastic drugs for previous disorder  10. Other concomitant tumor disease and/or tumor disease in the past 5 years (except basalioma of the skin and carcinoma in situ)  11. CNS involvement of lymphoma (intracerebral, meningeal, intraspinal)  12. Active serious infections not controlled by oral or intravenous antibiotics or anti-fungal  13. Any medical condition which in the opinion of the investigator places the subject at an unacceptably high risk for toxicities.  14. Non-conformity to eligibility criteria. |

CD: Cluster of differentiation; WHO: World Health Organization; B-NHL: B-cell Non-Hodgkin Lymphoma; DLBCL: Diffuse large B-cell lymphoma; EBV: Epstein-Barr virus; ALK: Anaplastic lymphoma kinase; IPI: international prognostic index; CIRS: Cumulative Illness Rating Scale; CHOP: Cyclophosphamide, Doxorubicin, Vincristine, Prednisolone; ECOG: Eastern Cooperative Oncology Group performance score; CCS: Canadian cardiac society; NYHA: New York Heart Association; FEV1: forced expiratory volume in 1 second; HIV: human immunodeficiency virus;

**Supplemental Table 2: Secondary neoplasia (n=4).**

**
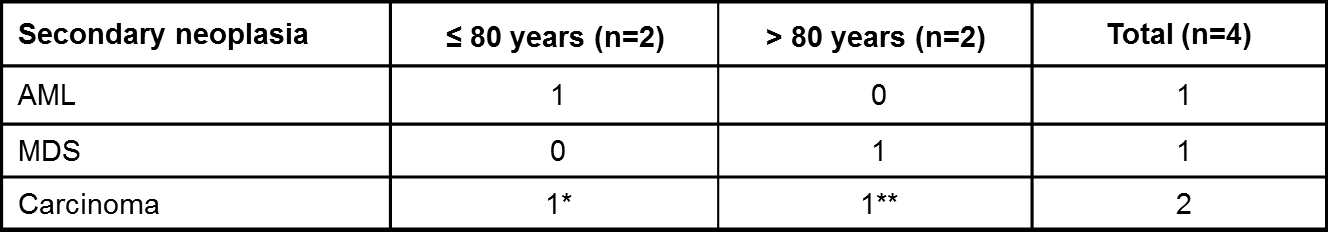
**

AML: Acute myeloid leukemia; MDS: Myelodysplatic Syndrome; * bronchial carcinoma, ** renal cell carcinoma

**Supplemental Table 3: Characteristics of B-R-ENDA patients aged > 80 years (n=39) and RICOVER-60 patients (6xCHOP-14+8xR) aged 76 – 80 years (n=40).**


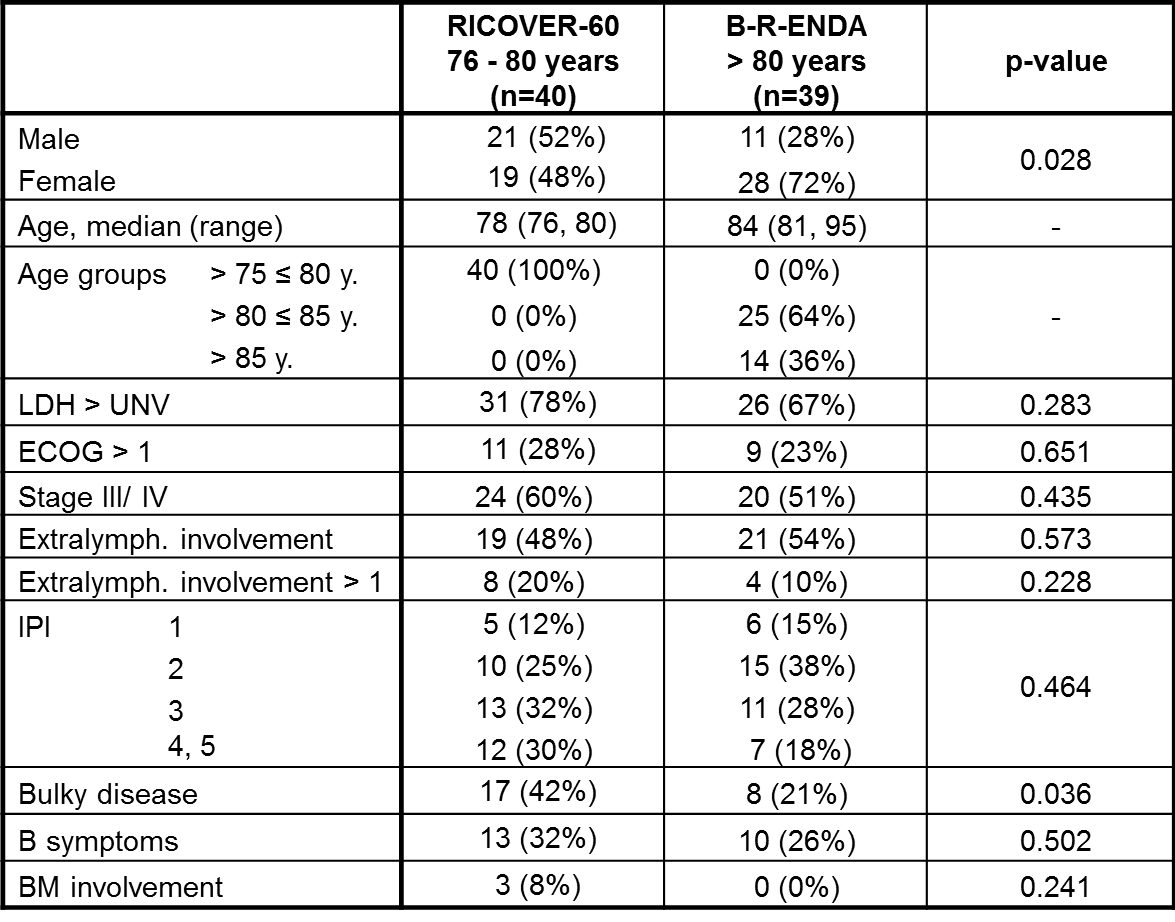


LDH: lactate dehydrogenase; UNV: upper limit of normal value; ECOG: Eastern Cooperative Oncology Group performance score; IPI: international prognostic index; Bulky disease: ≥ 7.5cm lymphoma mass; BM: Bone marrow; Bone marrow involvement is counted as extralymphatic involvement, spleen and Waldeyer Ring are counted as lymphatic involvement.

**Supplemental Table 4: Contributing centers.**

| **Study Center** | **Local PI** | **City** |
| --- | --- | --- |
| Klinikum Magdeburg gGmbH | C. Kahl | Magdeburg |
| Universitätsklinikum Ulm | A. Viardot | Ulm |
| Onkologische Gemeinschaftspraxis | G. Prange-Krex | Dresden |
| Charité Campus Benjamin Franklin | A. Korfel | Berlin |
| Gemeinschaftspraxis Hämatologie - Onkologie | T. Illmer | Dresden |
| Universitätsklinikum Jena | U. Wedding | Jena |
| Klinikum der Ludwigs-Maximilians-Universität München Campus Großhadern | M. Dreyling | München |
| Klinikum Nürnberg | J. Birkmann | Nürnberg |
| Gemeinschaftspraxis für Hämatologie und Onkologie | D. Reichert | Westerstede |
| Vivantes Klinikum Neukölln | M. De Wit | Berlin |
| Klinik für Hämatologie und Med. Onkologie Göttingen | L. Trümper | Göttingen |
| Onkologische Gemeinschaftspraxis | H.-J. Hurtz | Halle |
| Asklepios Klinik St. Georg | N. Schmitz | Hamburg |
| Klinikum Lippe-Lemgo GmbH | F. Hartmann | Lemgo |
| Onkologisches Studienzentrum Darmstadt | M. Rieger | Darmstadt |
| Praxis für interdisziplinäre Onkologie & Hämatologie | M. Zaiss | Freiburg |
| Universitätsklinikum Heidelberg | M. Witzens-Harig | Heidelberg |
| OncoResearch Lerchenfeld GmbH | V. Böhme | Hamburg |
| Universitätsklinikum des Saarlandes | M. Pfreundschuh | Homburg |
| Onkologische Schwerpunktpraxis Dres. Hansen & Reeb | R. Hansen | Kaiserslautern |
| Onkologische Gemeinschaftspraxis Dres. Söling/Siehl | U. Söling | Kassel |
| Klinikum Landshut gemeinnützige GmbH | B. Kempf | Landshut |
| Onkologische Praxis Oldenburg | B. Kühn | Oldenburg |
| Paracelsus-Klinik Osnabrück | W. März | Osnabrück |

**Supplemental Figure 1: Flow chart.**

Within the run-in phase (patient 1 to 20) patients received Rituximab 375 mg/m² IV (2^nd^ to 8^th^ application), within the main phase (patient 21 up to the end of recruitment) patients received Rituximab 1400 mg SC absolute (2^nd^ to 8^th^ application).

Diagnosis

**Prephase treatment**

d-7 to d-1 Prednisolone 100mg

d-3 Rituximab 375 mg/m^2^ IV (1^st^ application)

**Ordering of study**

**medication**

**during screening**

**Registration**

**possible until d-4**

**3x R-Benda**

D: day; IV: intra venous; R: Rituximab; Benda: Bendamustine; RE: restaging; PD: progressive disease; CR: complete remission; PR: partial remission; SD: stable disease; SC: subcutaneously; Gy: gray; bulky disease: ≥ 7.5cm lymphoma mass.

**Interim Staging RE1**

PD

CR, PR, SD

End of

study

treatment

**3x R-Benda**

**Final Restaging RE2**

PR, SD

CR

**39,6 Gy Rx only if residual**

**lesions of initial bulky disease**

**Restaging RE3**

**Follow-up**

d1 Rituximab 375mg/m^2^ IV or 1400 mg SC (5^th^ to 7^th^ application)

d1-2 Bendamustine 90mg/m^2^ IV

**1 x Rituximab 375 mg/m^2^ IV or 1400 mg SC** (8^th^ application)

d1 Rituximab 375mg/m^2^ IV or 1400 mg SC (2^nd^ to 4^th^ application)

d1-2 Bendamustine 90mg/m^2^ IV (first cycle day 2 and 3)

**Supplemental Figure 2: Duration and absolute dose of Bendamustine therapy.**

**
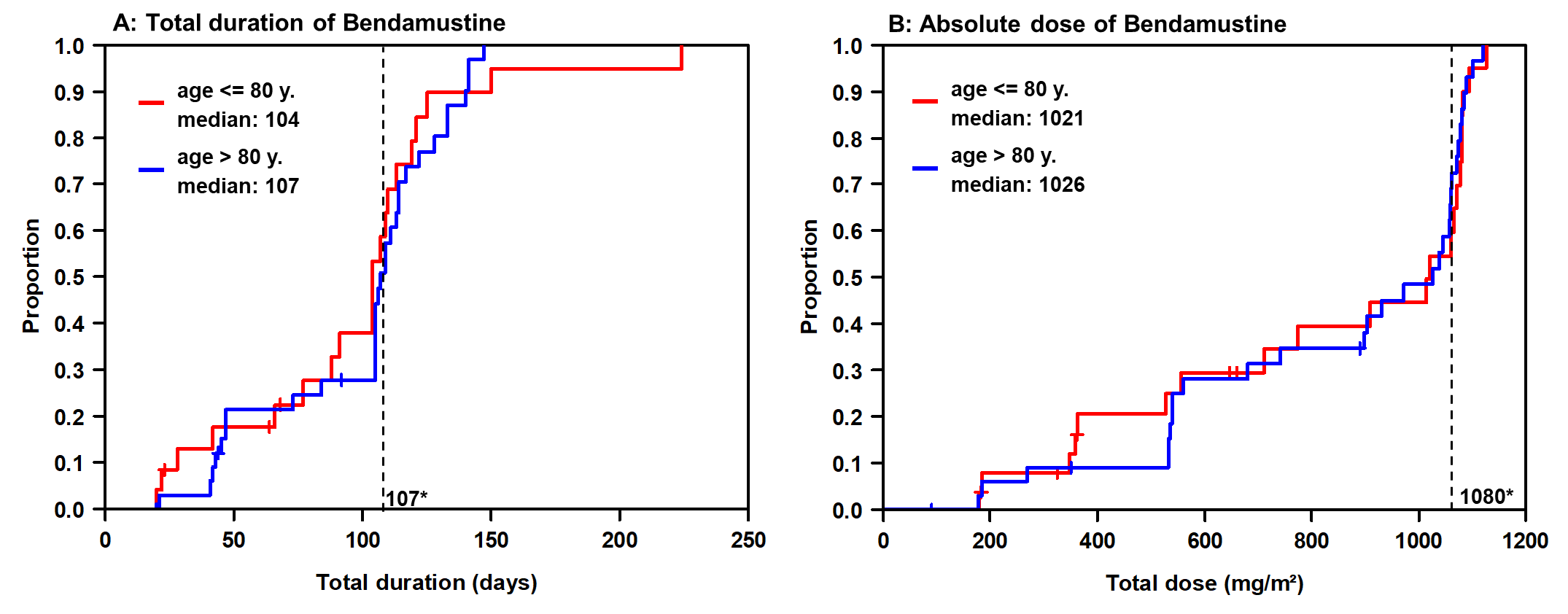
**

*(A) planned duration for Bendamustine: d1 (d1 of cycle 1) to d107 (d2 of cyle 6); only patients with at least two doses are included

* (B) planned absolute dose of Bendamustine (90mg/m² d1 and d2 for 6 cycles)

**Supplemental Figure 3: EFS (A), PFS (B) and OS (C) according to IPI 1, 2 (n=21) and 3-5 (n=18) for B-R-ENDA patients > 80 years of age.**

**A B C**


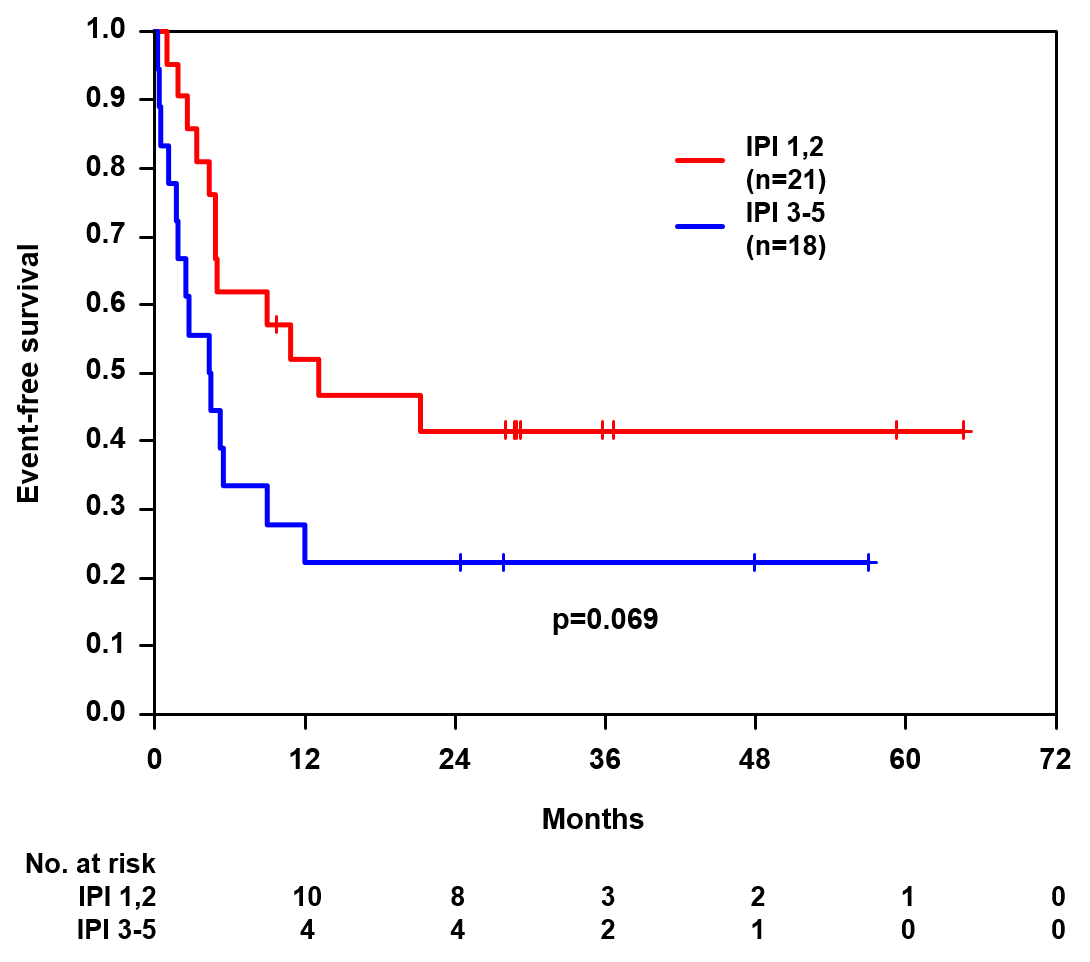

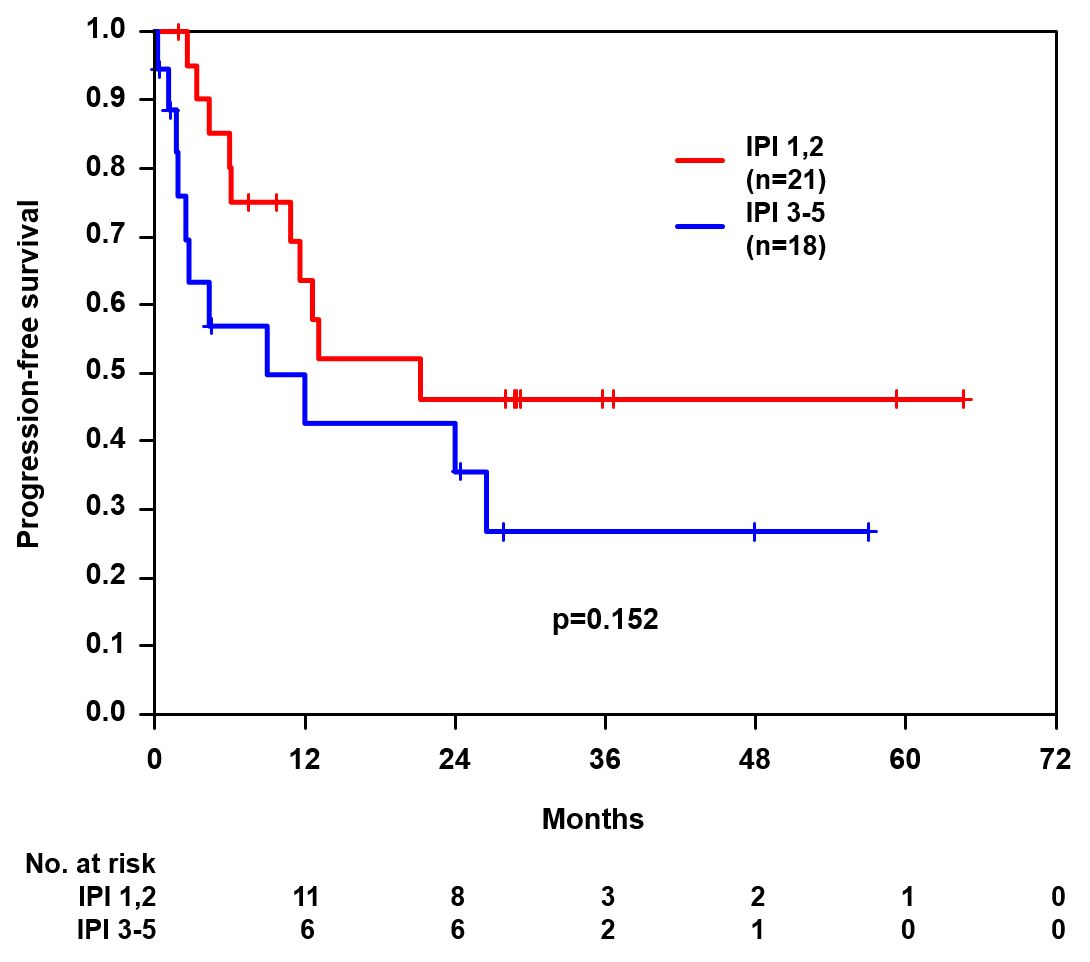

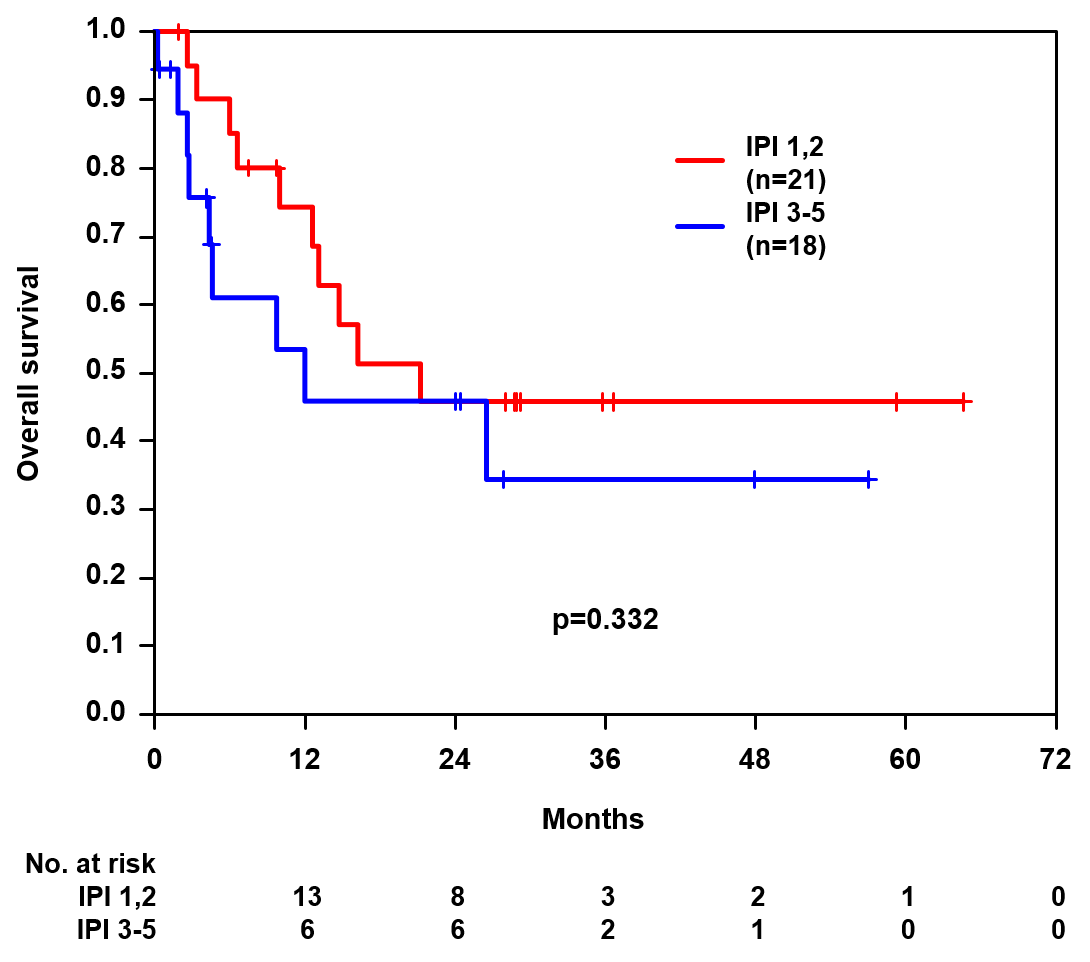


EFS: Event-free survival; PFS: progression-free survival; OS: overall survival; IPI: international prognostic index.

**Supplemental Figure 4: EFS (A), PFS (B) and OS (C) according to CIRS ≤ 6 (n=17) and CIRS > 6 (n=22) for B-R-ENDA patients > 80 years of age.**

**A B C**


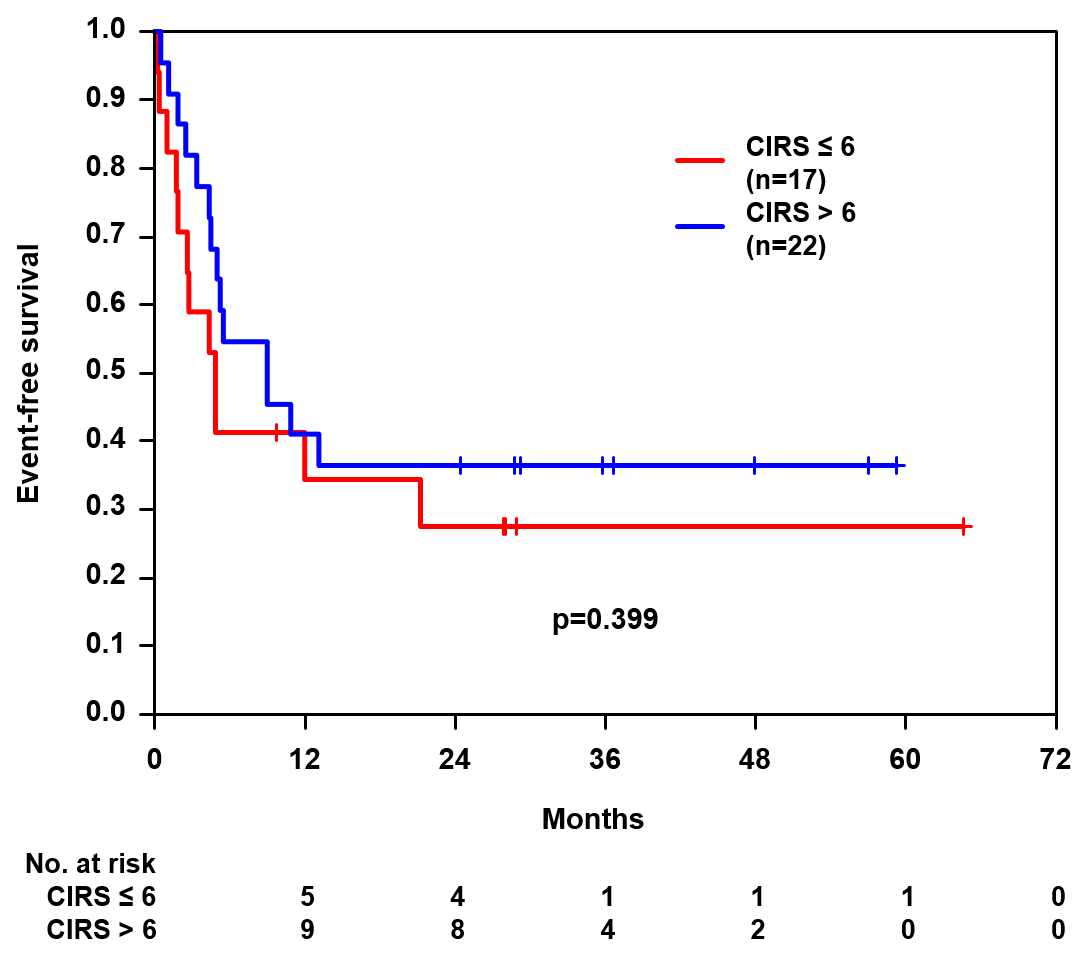

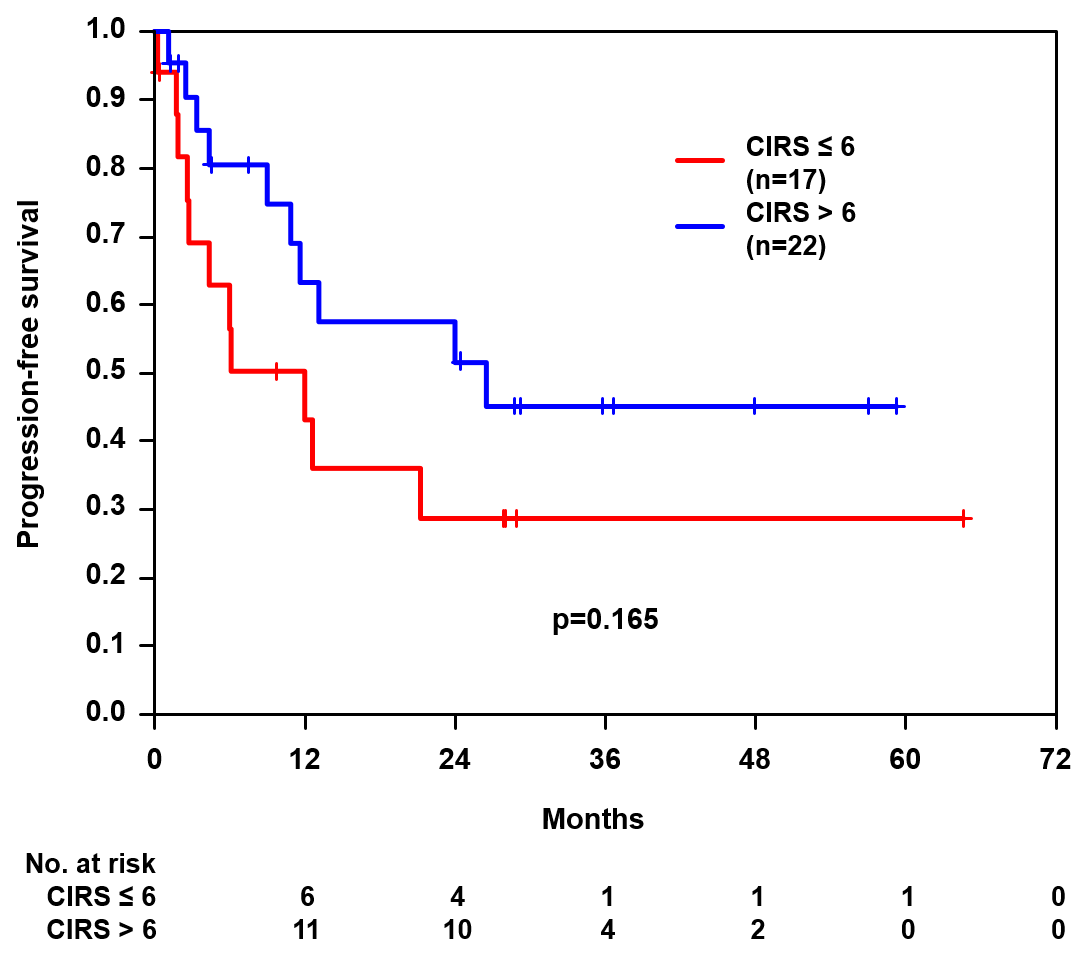

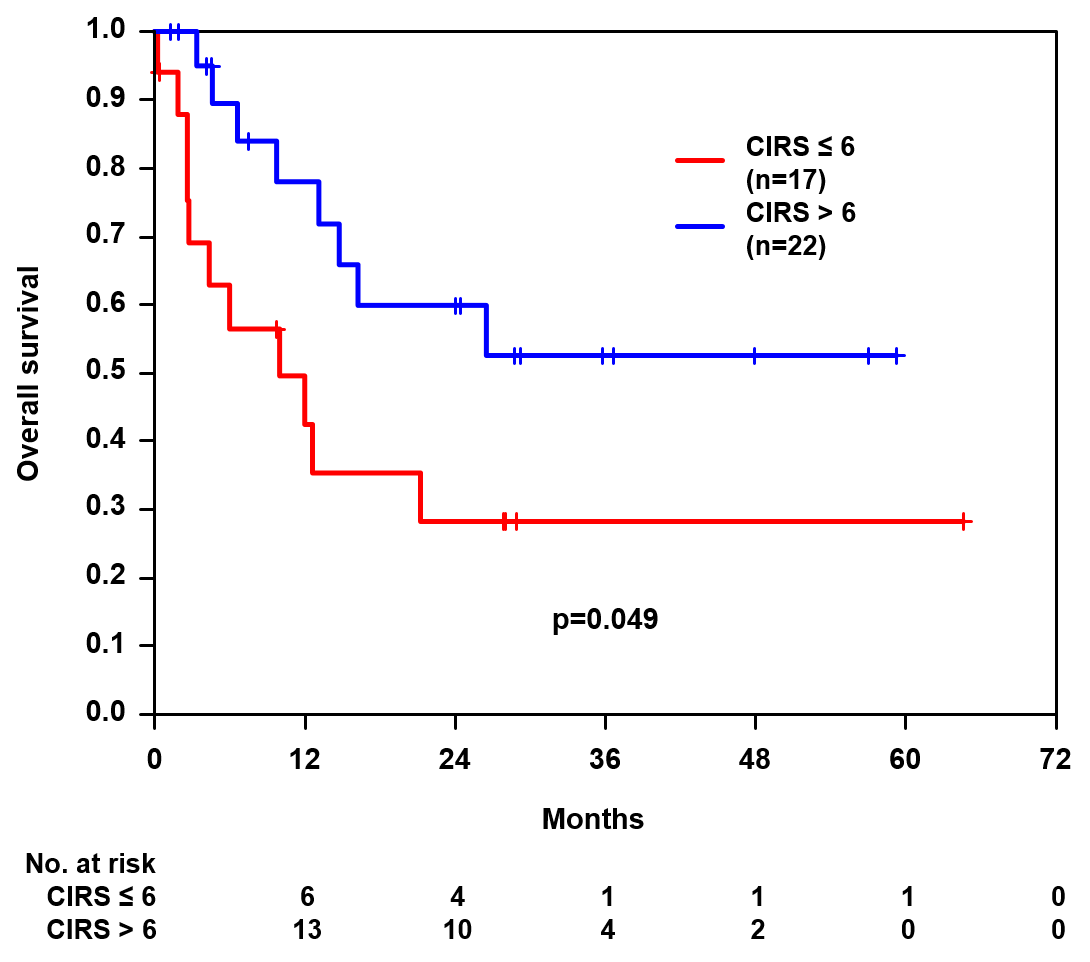


EFS: Event-free survival; PFS: progression-free survival; OS: overall survival; CIRS: Cumulative Illness Rating Scale.

**Supplemental Figure 5: EFS (A), PFS (B) and OS (C) according to G8 ≥ 14 (n=12) and G8 < 14 (n=27) for B-R-ENDA patients > 80 years of age.**

**A B C**


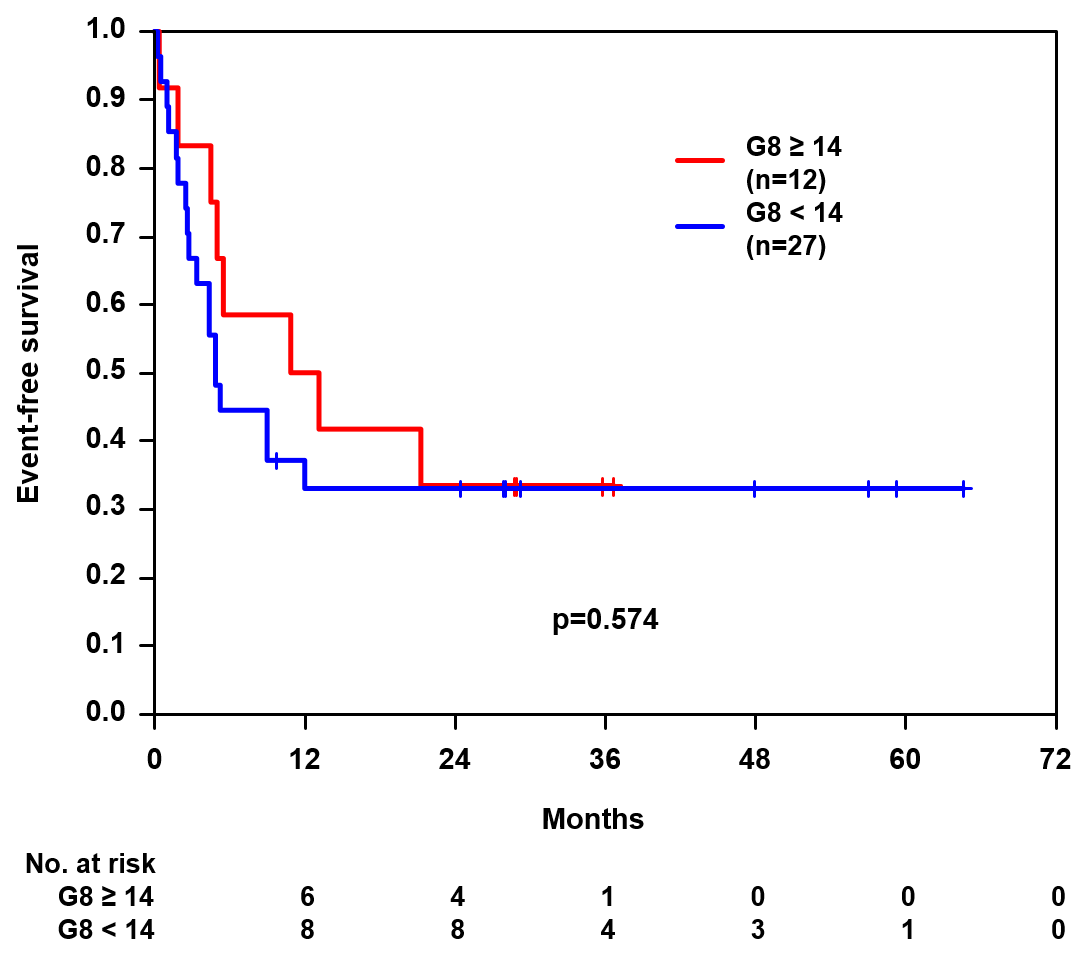

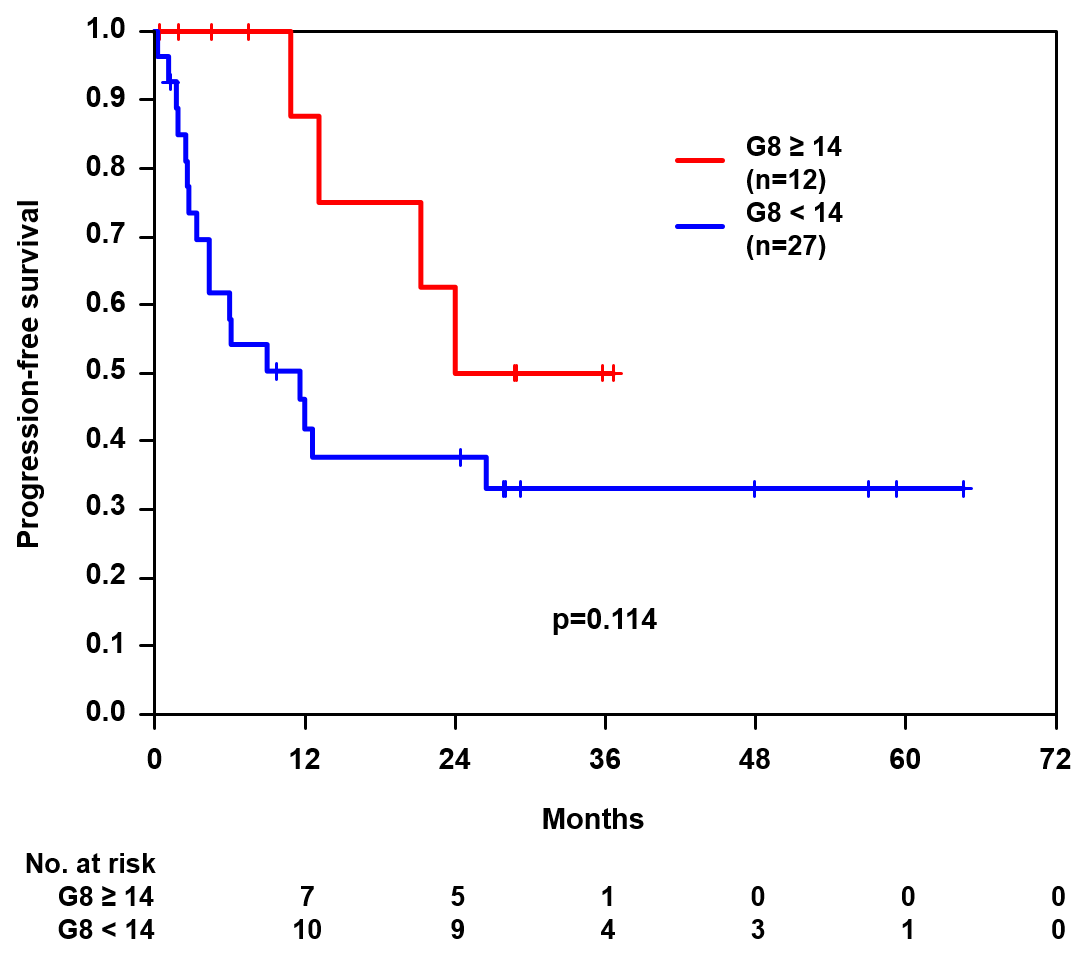

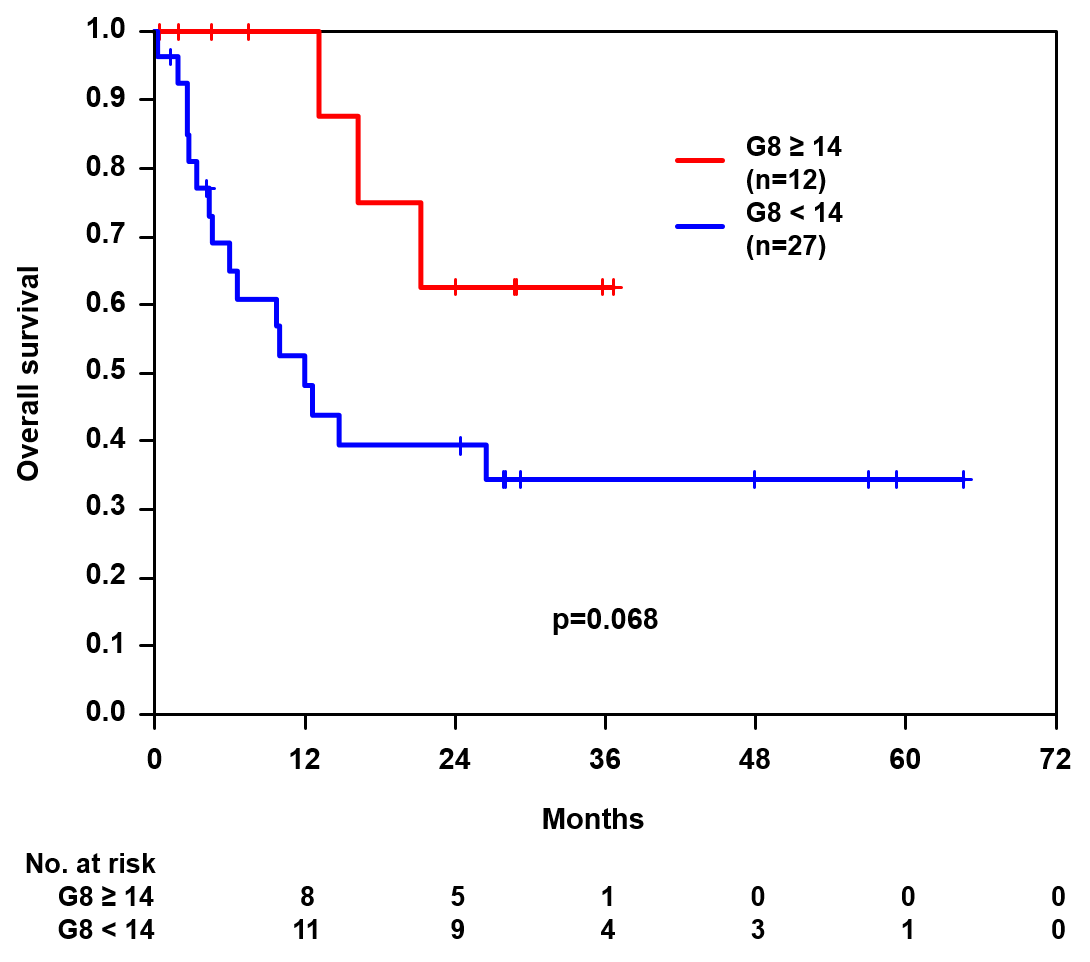


EFS: Event-free survival; PFS: progression-free survival; OS: overall survival; G8: Geriatric Screening Scale G8^27^.

**Supplemental Figure 6: EFS (A), PFS (B) and OS (C) according to IADL = 8 (n=16) and IADL < 8 (n=21) for B-R-ENDA patients > 80 years of age (two missing values for IADL).**

**A B C**


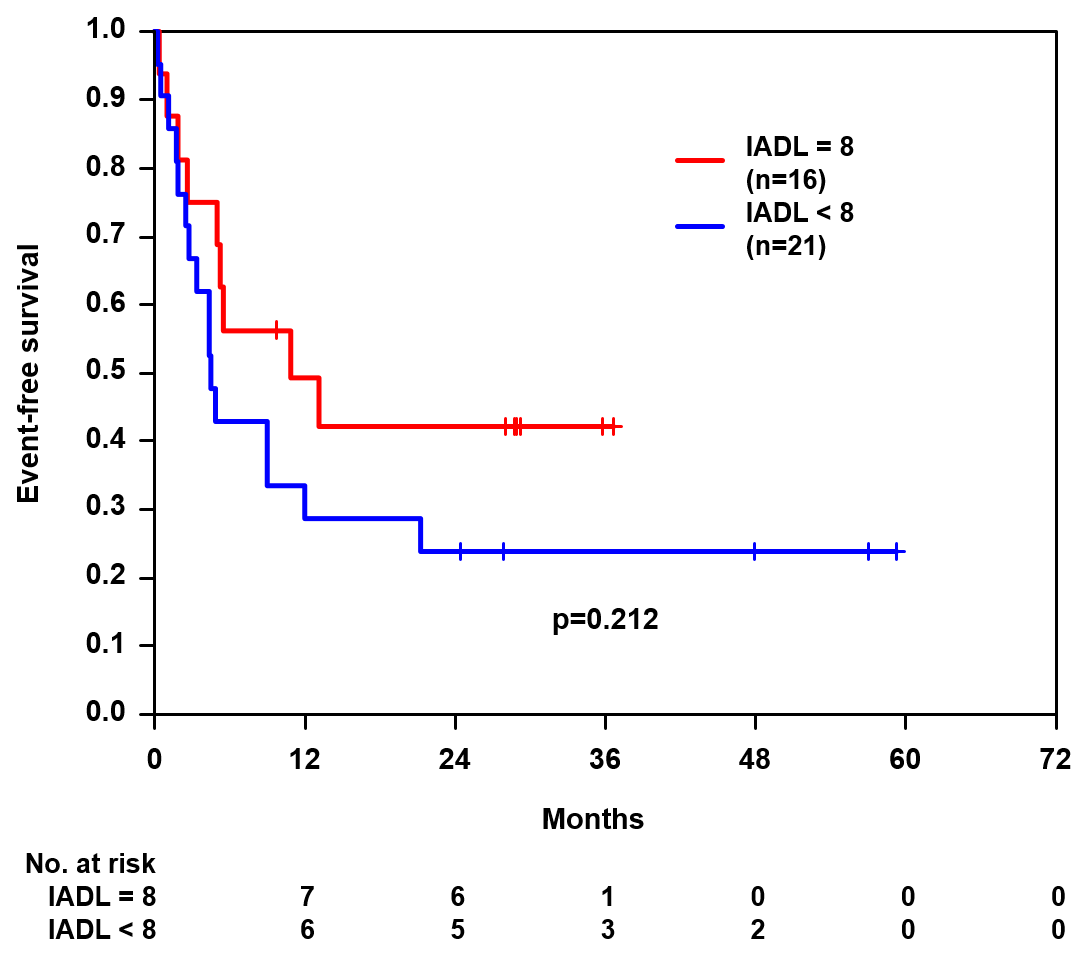

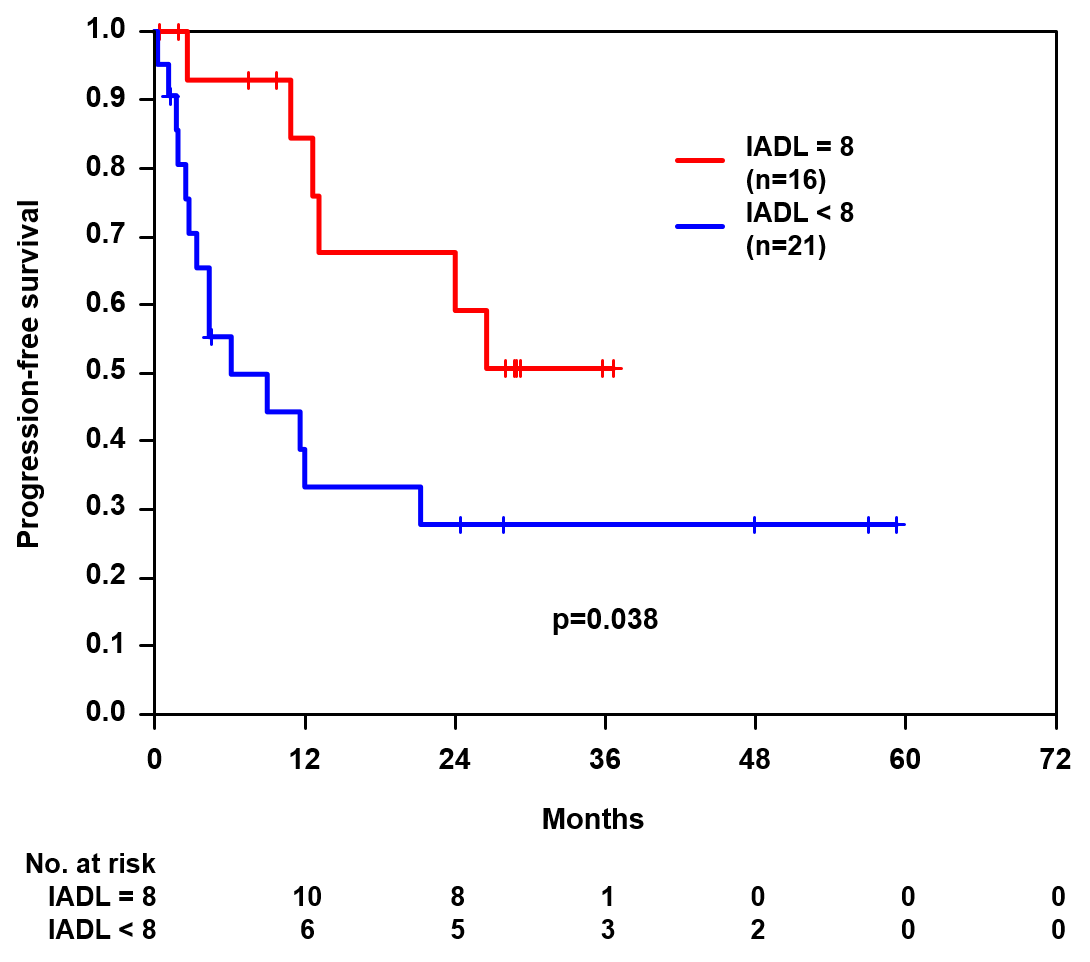

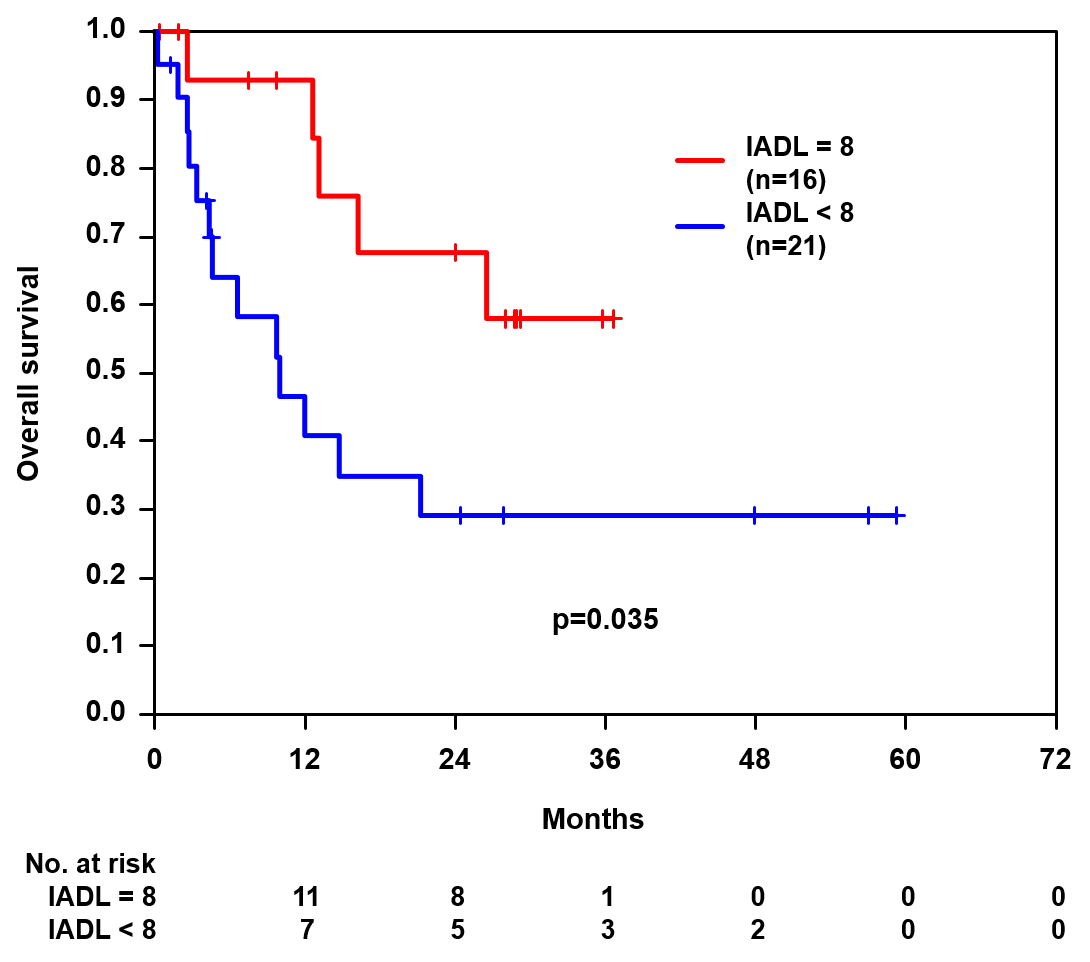


EFS: Event-free survival; PFS: progression-free survival; OS: overall survival; IADL: instrumental activities of daily life.

**Supplemental Figure 7: EFS (A), PFS (B) and OS (C) according to Quality of Life (EORTC) ≥ 50 (n=19) and Quality of Life (EORTC) < 50 (n=9) for B-R-ENDA patients > 80 years of age.**

**A B C**


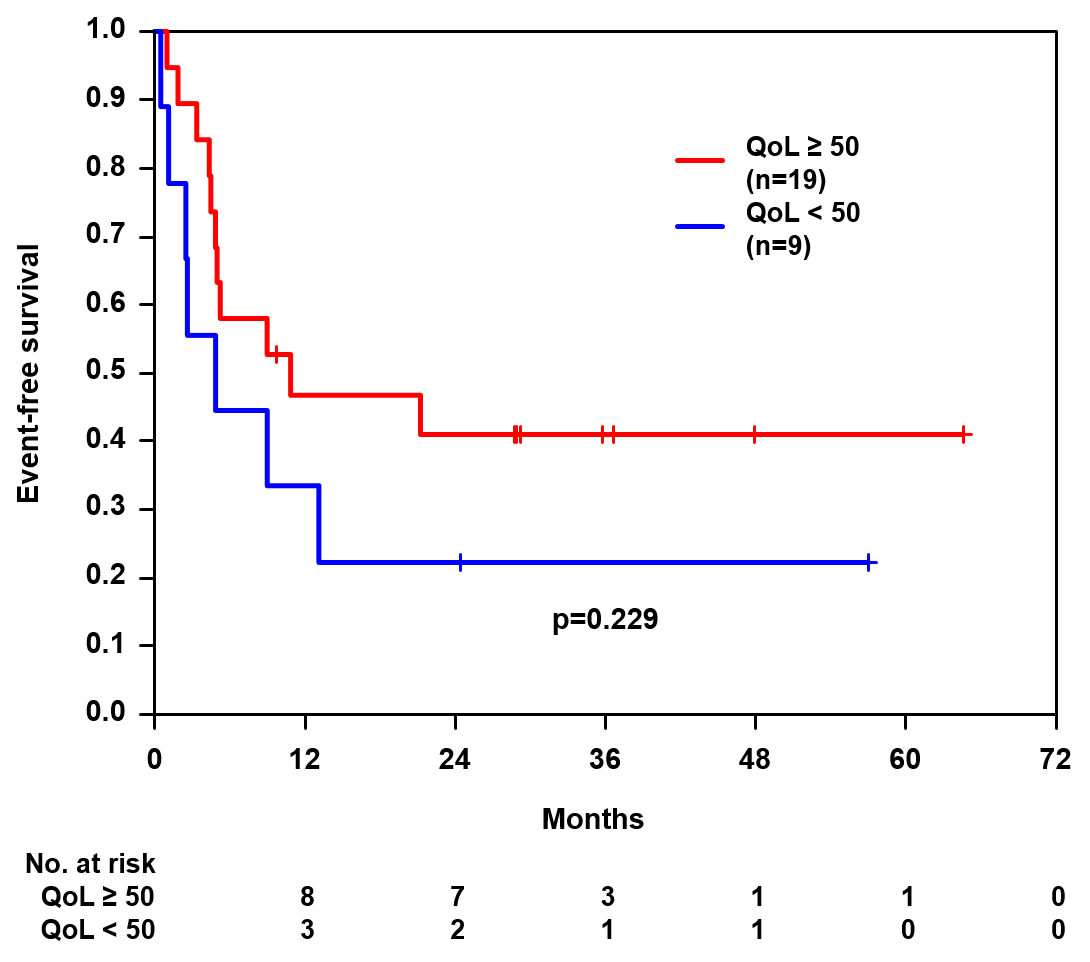

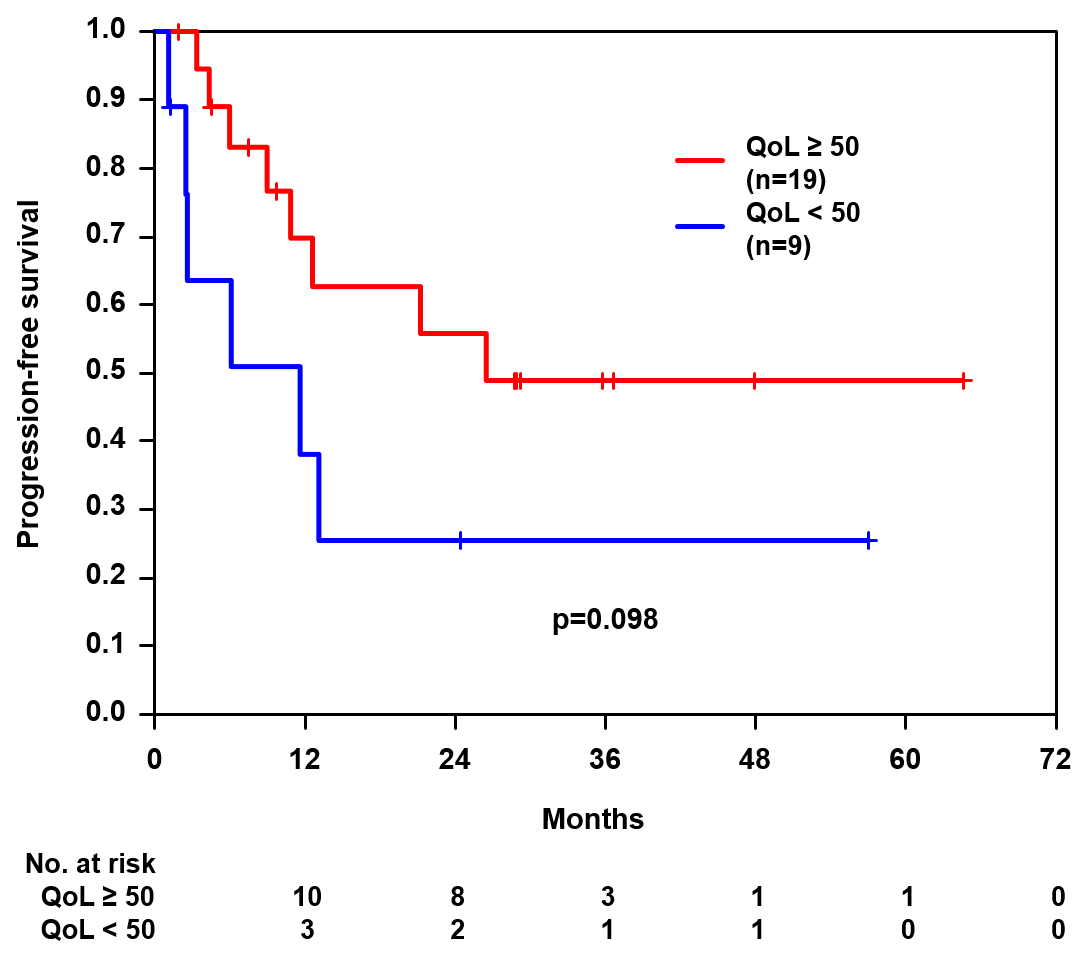

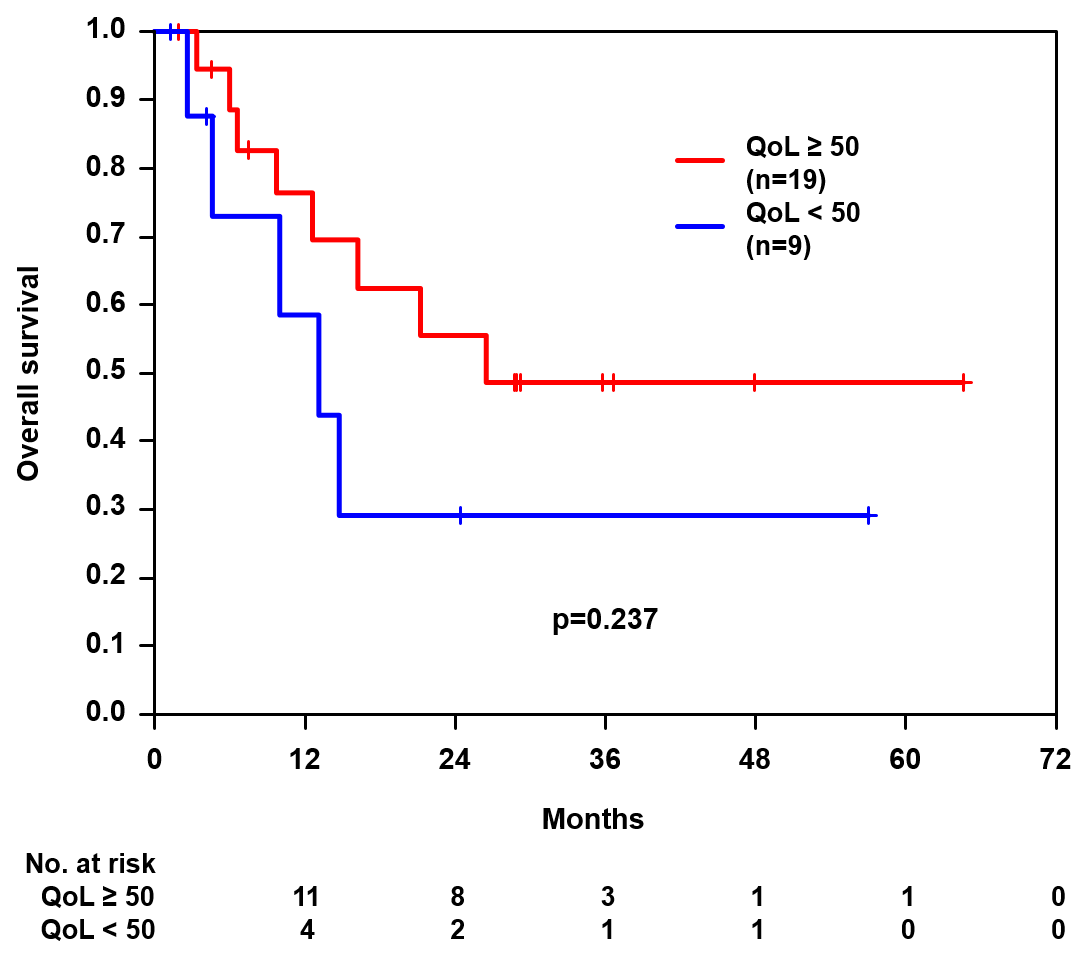


**Supplemental Figure 8: Event-free-survival (A, D), progression-free survival (B, E) and overall survival (C, F) of B-R-ENDA patients aged > 80 years (n=39) and RICOVER-60 patients (6xCHOP-14+8xR) aged 76 – 80 years (n=40) according to low (A – C) and high (D – F) IPI scores.**

**A B C**


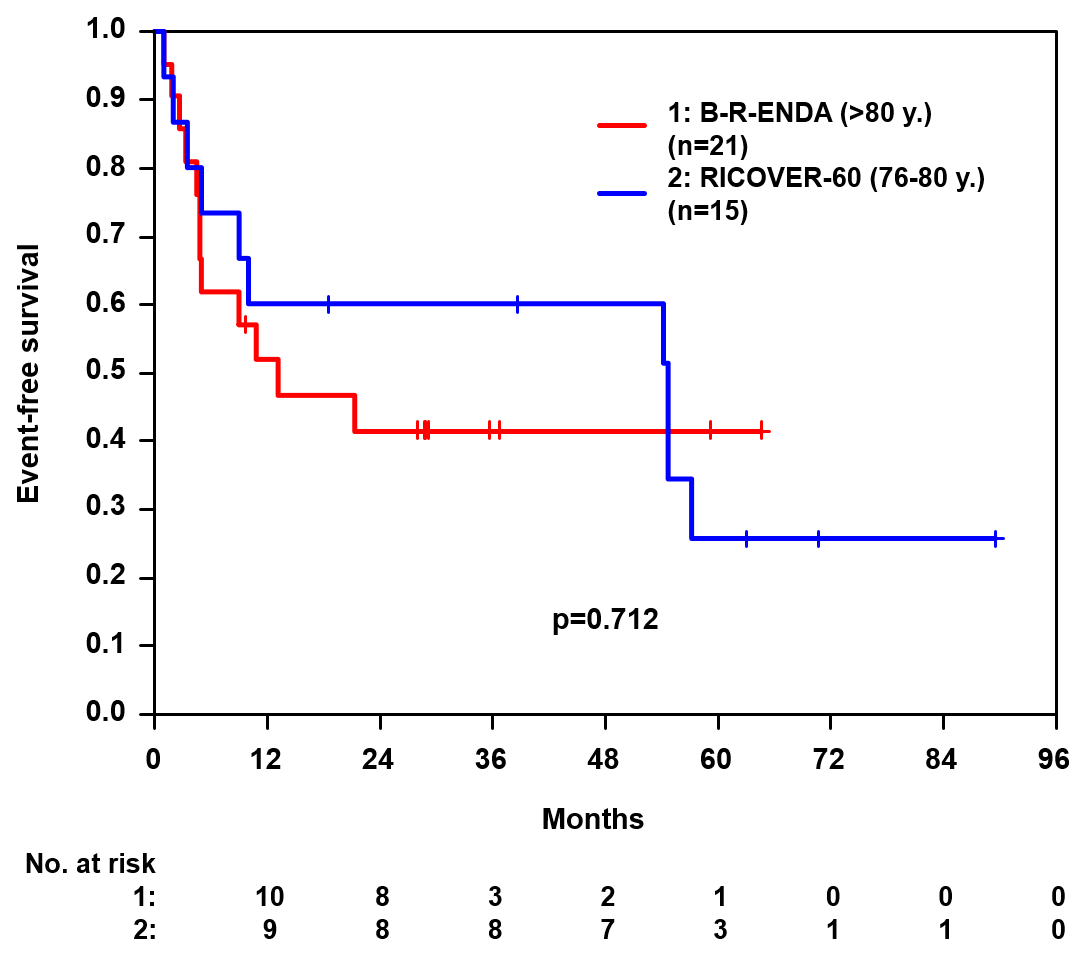

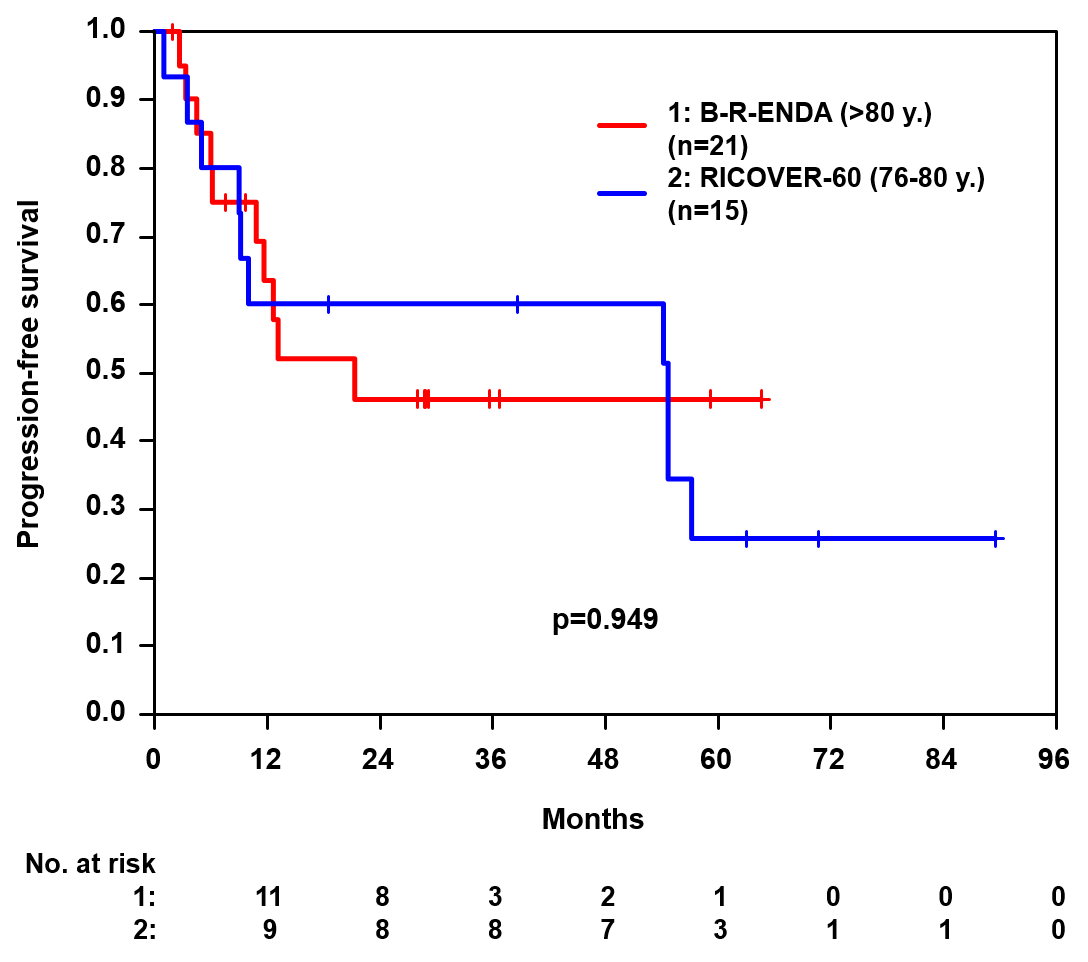

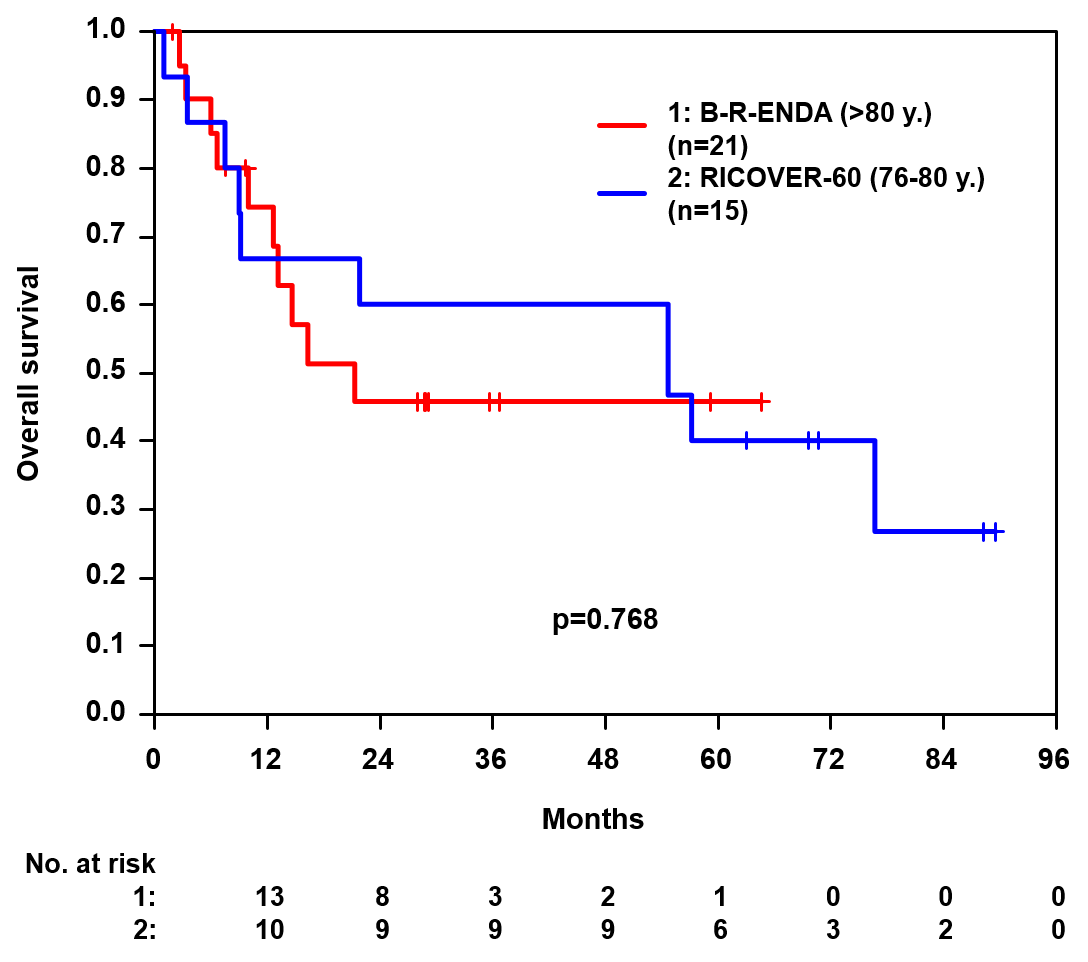


**D E F**


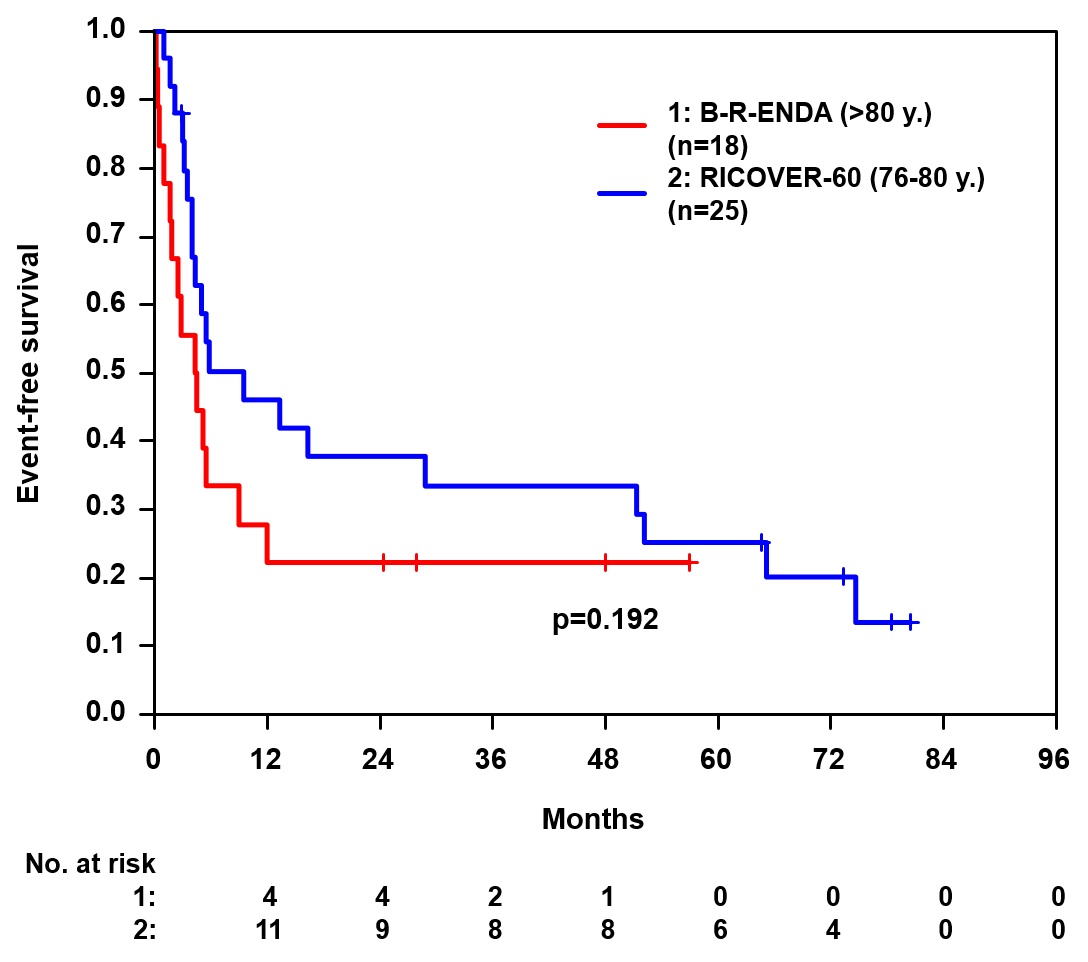

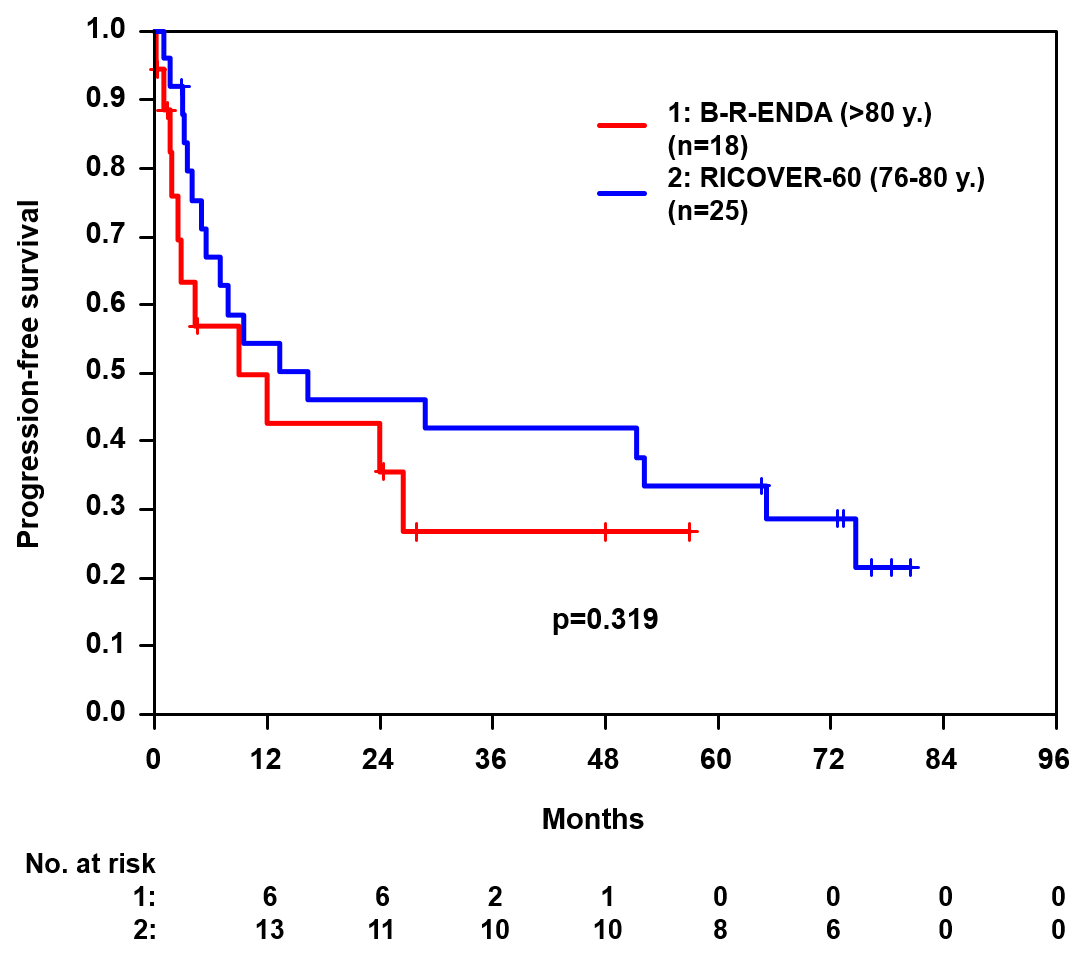

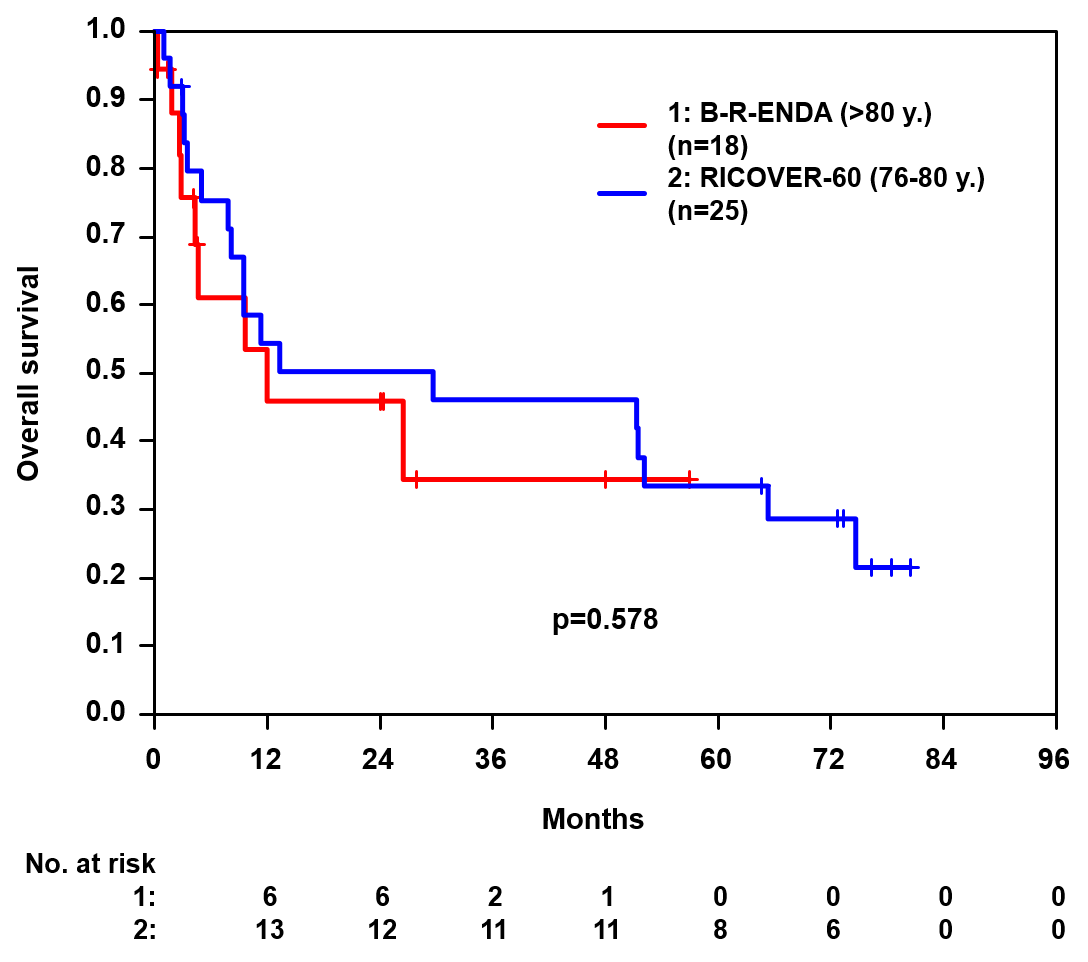

Supplement: Supplementary file 1 [file hs9-6-e808-s001.docx]
